# Characterization and profiling of bacteriocin-like substances produced by lactic acid bacteria from cheese samples

**DOI:** 10.1099/acmi.0.000234

**Published:** 2021-06-08

**Authors:** Sadia Afrin, Mohammad Amirul Hoque, Ashish Kumar Sarker, Mohammed A Satter, Mohammad Nazrul Islam Bhuiyan

**Affiliations:** ^1^​ Industrial Microbiology Laboratory, Institute of Food Science and Technology (IFST), Bangladesh Council of Scientific and Industrial Research (BCSIR), Dhaka-1205, Bangladesh; ^2^​ BCSIR Laboratories, Dhaka-1205, Bangladesh Council of Scientific and Industrial Research (BCSIR), Bangladesh

**Keywords:** antimicrobial activity, bacteriocin, cheese, lactic acid bacteria

## Abstract

Bacteriocins have become biological weapons against harmful food pathogens and have attracted interest as tools for biopreservation. The aim of this study was to isolate, identify and characterize lactic acid bacterial (LAB) strains from cheese samples, partially purify potential bacteriocins and characterize their antimicrobial activity against pathogens. Bacteriocin-producing organisms were screened by Agar spot assay test. Initially, 25 LAB isolates were isolated from the cheese samples and identified as *Lactobacillius* spp., among them five strains were able to produce bacteriocin whose antimicrobial activates were analysed by agar-well-diffusion assay test against pathogenic organisms. *Bacillus subtilis*, *Bacillus cereus*, *Staphylococcus aureus*, *Streptococcus thermophillus* and *Listeria monocytogens* were inhibited, while *Enterococcus faecalis*, *Salmonella typhi*, *Escherichia coli* and *Pseudomonas aeruginosa* were resistant to the antimicrobial substances from LAB isolates. For optimal production of bacteriocin, LAB broth cultures were harvested at exponential phase. The molecular weights of the bacteriocins are between 7.0–15.0 kDa. The bacteriocins were characterized on the basis of their sensitivity to heat, pH, enzymes, NaCl and treatments with organic solvents. These results revealed that the bacteriocins from *Lactobacillius* spp. isolated from the cheese might have potential antimicrobial properties and give new insight in the development of bio-preservative agents to prevent and control pathogenic bacterial infection.

## Introduction

Cheese is a milk product that is produced by eliminating water from curd, consisting of fats, milk proteins and other food ingredients as well as elements conferring distinct aroma and flavour [1]. In the cheese-making process, the milk proteins change and at the same time its sugar composition is also modified. In particular, the milk sugar lactose is transformed into lactic acid via a microbial fermentation process and consequently contributes to the ripening process of cheese [2, 3]. Lactic acid bacteria (LAB) are a pioneer group of micro-organisms that actively take part in the fermentation process of different food products. LABs possess a complicated enzyme system due to amino acid requirement and contribute to cheese flavour. As a result, LABs play a critical role during the cheese-making process [4]. LABs belong to diverse phylogenetic groups, usually Gram-positive and have a beneficial effect on human and animal health [5]. LABs including *Lactobacillus* spp. are of increasing interest for use in the prevention and control of diverse diseases. These organisms have been commonly employed as probiotics [6, 7]. Since LABs have a valuable effect on the human gastrointestinal tract, presently they are of significant interest in industrial and medical application. LABs are extensively used in food processing because of their ability to increase the organoleptic characteristics of food, with a parallel decrease of pH that inhibits undesirable micro flora [8, 9]. In addition, LABs can synthesize and excrete some antimicrobial substances (lactic acid, diacetyl, hydrogen peroxide, bacteriocins and so on), which are able to restrain both Gram-positive and Gram-negative pathogenic bacteria [10, 11]. Bacteriocins from LABs are ribosomal synthesized lower-molecular weight proteins or peptides with potential use in natural food preservation due to their bactericidal or bacteriostatic effects. Among the diverse bacteriocins described to date, LAB bacteriocins have concerned a great deal of attention as their producers are a ‘generally recognized as safe (GRAS)’ micro-organism [12–14]. Presently, extensive study has been performed with bacteriocins and bacteriocin producing LAB strains. LAB bacteriocins are divided into three major classes on the basis of structure and characteristics. Class-I consists of bacteriocins (<5 kDa) usually referred to as lantibiotics, containing one or more modified amino acid residues lanthionine, b-methyllanthionine, dehydroalanine, dehydrobutyrine and/or d-alanine. Class-II contains small (<10 kDa) heat-stable non-lanthionine peptides and divided into four groups: Class-IIa consists of *Listeria* active peptides with an N-terminal consensus sequence, class-IIb are the two-peptide bacteriocins, class-IIc contains cyclic bacteriocins whose N- and C-termini are covalently linked, and class-IId contains the one peptide non-cyclic bacteriocins that show no sequence similarity to the pediocin-like bacteriocins. Class-III consists of large heat-labile protein bacteriocins (more than 30 kDa). According to Klaenhammer’s classification scheme, most of the bacteriocins produced by *Lactobacillus* spp. are categorized as class-II type bacteriocins, with a few exceptions [15–17]. Moreover [18], verified that the bacteriocin from *L. acidophilus* KS400 has strong antimicrobial capability against relevant urogenital pathogens. The best studied bacteriocin ‘nisin A’ produced by *L. lactis*, is approved as safe by the World Health Organization (WHO) and the Food and Agriculture Organization (FAO), and used as a food preservative all over the world [19–21]. The bacteriocins from *Lactobacillus* spp. are proven to be safe for human consumption, to not modify the nutritional properties of the food, to be effective at very low concentration and active under refrigerated storage conditions [11, 22]. In addition to their applications in food-related industries for the enhancement of food quality and safety, the bacteriocin from LAB strains have been proposed to modulate the immune system [23, 24]. To understand the promising significance of bacteriocins, five novel *Lactobacillus* spp. were isolated from cheese samples. The objectives of this study were to isolate bacteriocins from this *Lactobacillus* spp. and evaluate their antimicrobial spectrum against a range of bacteria. Furthermore, the inhibition patterns of bacteriocins from *Lactobacillus* spp. were performed in response to a different temperature, pH, salinity (NaCl) as well as enzymes and organic solvents. The obtained results give new insight in the development of bio-preservatives agent to prevent and control the pathogenic bacterial infection.

## Methods

### Collection and enrichment of samples

A total of eight local cheese samples, made of cow’s milk were collected randomly from different places in Bangladesh in 2018. Each sample (25 g) dissolved separately into 225 ml of sterile buffer peptone water and blended with a Stomacher machine (Stomacher 400, Seward) for 5 min. The samples were then enriched in *Lactobacillus* de Man, Ragosa Sharpe (MRS) broth medium (Hi-Media, India) for 24 h at 37 °C.

### Isolation of LAB

The enriched cheese samples in MRS broth were streaked on to the MRS agar plates and were incubated in anaerobic jar at 37 °C for 72 h. The assumed *Lactobacillus* species were purified with repeated streak plate technique. Microscopic observation and Gram reaction were performed for the isolates of 18 h culture from MRS agar plates. The selected isolates were maintained as stock cultures frozen at −20 °C in 15 % (v/v) glycerol. Strains were propagated twice before used in experiments.

### Identification by morphological and biochemical characteristics

Identification of the *Lactobacilli* was performed according to their morphological, physiological and biochemical characteristics [12, 25]. The preferred biochemical tests (Simmon’s Citrate, Indole, Methyl Red, Voges Proskauer, Oxidase and Catalase) were performed as recommended in *Bergey’s Manual of Determinative Bacteriology* [26]. The identified genus *Lactobacillus* was further classified to the species level based on their ability to ferment sugars (arbinose, cellobiose, fructose, galactose, lactose, maltose, manitol, mannose, raffinose, rhamnose, sucrose and xylose) [27].

### Identification by BIOLOG system

For the species-level identification of antagonistic isolates, BIOLOG identification system was applied (BIOLOG, USA) base on the utilization of 71 carbon sources and 23 chemical sensitivity assays in GEN III microplate test panels. The isolates were cultured on Biolog universal growth (BUG) agar medium. All microplates and inoculating fluid were pre-warmed at 37 °C for 30 mins. After 18 h incubation the inoculum of antagonistic bacterial isolates were added to the inoculating fluid-A for obtaining the desired turbidity, which is (90–98)% T because the target cell density must be in the range of (90–98)% T for protocol-A of GEN III Microbial ID 20 assay techniques on GEN III microplate. The cell suspension was then poured into the multichannel pipette reservoir and the tips of multi-channel. A repeating pipettor was filled by drawing up the cell suspension from the reservoir and all 96 wells were inoculated with accurately 100 µl of bacterial suspension. The microplate was then covered with its lid and incubates at 37 °C for 18 to 24 h. After incubation, the microplate was placed into the Micro Station Reader for analysis to obtain ID result and the result was given through comparing with database using the software programme MicroLog 4.20.05 (BIOLOG, USA). The scope of the 96 assay reactions, coupled with sophisticated interpretation software, delivers a high level of accuracy that is comparable to molecular methods.

### Extraction of crude bacteriocin-like substances

Selected *Lactobacillus* isolates were grown in MRS broth within a shaking incubator at 125 r.p.m. and 37 °C for 48 h in anaerobic condition. The cultures were then collected into a centrifuge tube and centrifuged at 10 000 ***g*** for 15 min at 4 °C, and was adjusted to pH 7.0 by means of 1M NaOH to exclude the antimicrobial effect of organic acid. The cell-free supernatant was filtered with 0.2 µm membranes filter (Millipore) and used as crude bacteriocin [4, 28, 29]. Extracted bacteriocin was stored at −20, 4 and 37 °C. At different time intervals, samples were taken from the stored material to determine bacteriocin activity [30].

### Partial purification of bacteriocins

The bacterial pellet was discarded and the cell-free culture supernatant (crude bacteriocin) was saturated with 70 % ammonium sulphate and stored at 4 °C for the precipitation of proteins. The pellet was collected after centrifugation at 10 000 ***g*** at 4 °C for 30 min. The pellet was dissolved in phosphate buffer (0.1M, pH 7.0) and dialyzed against the same buffer at 4 °C overnight. The resulting filtrates were used to evaluate antimicrobial activity [28, 29].

### Molecular weight determination

Protein concentration was estimated according to [10]. The molecular weight of the purified bacteriocin was determined by 12 % sodium dodecyl sulphate polyacrylamide gel electrophoresis (SDS-PAGE) (Bio-Rad, USA) as previously described by [31]. Electrophoresis was carried out at 20 mA until the tracker dye (Bromophenol blue) reached to the bottom of the gel. After electrophoresis at 20 mA, the gel was stained with Commassie Brilliant blue and destained by washing overnight with mixture of acetic acid, methyl alcohol and water. The molecular weights were estimated using SDS-PAGE protein molecular weight pre-stained markers (Promega, USA).

### Assessment of minimum inhibitory concentration (MIC) and minimum bactericidal concentration (MBC)

The MIC was determined as the lowest concentrations an antibacterial agent that entirely inhibits the bacterial growth and MBC is the lowest concentrations to kill a particular bacterium [32]. The broth micro-dilution methods according to CLSI guidelines was employed to determine the MIC and MBC [33]. Ciprofloxacin (500 mg) was used as the positive control and 10 % DMSO-soaked filter paper disc was used as the negative control. The plates were incubated at 37±2 °C for 18–24 h. Antimicrobial activity was assessed by measuring absorbance at 690 nm of wave length.

### Bacteriocin activity assay

The antibacterial activities of the extracted bacteriocin were determined using the well-diffusion method slightly modified using agar and gellan gum, respectively as described by [34]. Aliquots of the each bacteriocin sample (100 µl) were placed in 6 mm diameter wells that had been cut in agar plates/gellan gum plates previously seeded with the bacterial pathogen such as *Bacillus cereus*, *Bacillus subtilis*, *Staphylococcus aureus*, *Staphylococcus epidermidis*, *Enterococcus faecalis*, *Staphylococcus faecalis*, *Listeria monocytogenes*, *Streptococcus thermophilus*, *Clostridium* spp., *Pseudomonas aeruginosa*, *Vibrio parahemolyticus*, *Salmonella typhi*, *Shigella flexineri*, *Escherichia coli*, *Klebsiella* spp., *Serratia marcescens*, *Lactobacillus acidophilus*, *Lactobacillus brevis*, *Lactobacillus oris*, *Lactobacillus vaginalis* and *Lactobacillus gasseri*. These strains were previously isolated and identified in the Industrial Microbiology Laboratory, BCSIR. The plates were incubated at 37 °C for 24 h. After incubation, the diameters of the inhibition zone (*x*) were measured and calculated as follows: *x*=*D*–*d*, where *D* is the inhibition zone diameter and *d* is the well diameter. Antimicrobial activity (*x*) was characterized and classified based on the inhibition growth zone diameters and described as slight (*x*<4 mm diameter), medium (*x*=4–8 mm), high (*x*=8–12 mm) and very high (*x*>12 mm) [3, 6, 34].

### Thermal stability assay

To determine the effect of temperature 1.0 ml of partial purified bacteriocin was added into 5.0 ml of nutrient broth medium. Each test tube was then overlaid with paraffin oil to prevent evaporation and then heated at different temperatures (30, 40, 50, 60, 70, 80, 90 and 100 °C) for 20 min. Furthermore, to ensure the activity of bacteriocin at high autoclaving temperature the samples containing nutrient broth and bacteriocin were plugged with cotton and wrapped by aluminium foil and kept in an autoclave at 121 °C or 15 lbs pressure for 10 min. Agar-well-diffusion method was applied to evaluate the activities of above different heat-treated bacteriocin [19, 35].

### pH resistance and salinity (NaCl) stability assay

Bacteriocin (1.0 ml) was added into 5.0 ml of nutrient broth medium with varying pH value, ranging from 3.0 to 11.0 and incubated at 37 °C for 30 min. Each of the bacteriocin samples treated at different pH values was assayed against pathogenic bacteria by agar-well-diffusion method [36]. The activities of bacteriocin in nutrient broth medium containing different levels (1, 2, 3, 4, 5, 6 and 7 %) of NaCl at 37 °C for 30 min were determined by agar-well-diffusion method.

### Effect of enzymes, organic solvents and UV light

The proteolytic enzyme sensitivities of the purified bacteriocin produced by LAB species were tested individually by adding trypsin as illustrated by [37]. The effect of the enzymes (protease K, trypsin and amylase) on bacteriocin activity was studied by agar-well-diffusion method. Bacteriocins were treated with enzymes in the ratio of 1 : 1. Three preparations were used such as, enzyme control 1 (C1) contained 0.3 ml of phosphate buffer (0.5M, pH 7.0), enzyme control 2 (C2) contained 0.15 ml of bacteriocin and 0.15 ml of phosphate buffer (0.5M, pH 7.0), while for the enzyme reaction (ER) 0.15 ml of trypsin (0.25 mg ml^−1^) and 0.15 ml of purified bacteriocin were used. Reduction in zone size clearly indicated inactivation of bacteriocin (protein) due to the enzymes. All measurements were performed in triplicate and average values are reported. The bacteriocins from LAB isolates were placed in sterile glass ware and exposed to short-wave UV light at a distance of 30 cm. Times of exposure ranged from 0 to 5 min [38]. Within each time interval, bacteriocin activities were analysed by the agar-well-diffusion method using agar and gellan gum, respectively.

### Statistical analysis

All experiments were carried out in triplicate. Experimental data were analysed using one-way ANOVAs. Data were presented as the mean±sd for the indicated number of independently performed experiments.

## Results

### Isolation and morphological determination of LAB

From the cheese samples, a total of 30 colonies were initially presumed as *Lactobacillus* spp., on the basis of their morphological characteristics. The bacterial colonies were small with entire margins and white in colour. Under a light microscope the isolates were visible as either long rods (13 isolates) or short rods (17 isolates) and were Gram negative.

### Identification and characterization

Over the last two decades a variety of methods have been used for bacterial identification. In this study, the selected strains were initially identified based on physiological and biochemical characteristics and confirmed using the BIOLOG identification system.

### Identification by biochemical characteristics

Important biochemical tests were carried out for provisional identification. Most of the strains were unable to reduce nitrates, liquefy gelatin and were indole catalase and oxidase negative. A part of the isolates was able to utilize citrate. Sugar utilization is the important criteria for *Lactobacillus* identification. In this study, 12 different sugars were used for the identification of the isolates. Thus the sugar utilization patterns were compared with those given for *Lactobacillu*s species in *Bergey’s Manual of Determinative Bacteriology* [26]. The result indicated that 25 isolates were identified as *Lactobacillus* genera.

### Identification using BIOLOG system

The strains were correctly identified with BIOLOG system up to the species level. The BIOLOG system aims to provide a swift, convenient approach to bacteria identification with a database of 3000 species. The result indicates that among 25 (biochemically identified) isolates five isolates correctly identified up to species level (Table 1). Tests were carried out in triplicate.

**Table 1.** Micro-organism identification with BIOLOG

Strain name	ID (identification)	PROB (probability)	SIM (similarity index)	DIST (distance)
C1	* Lactobacillus plantarum *	0.937	0.542	1.152
C2	*Lactobacillus paracasei ss paracasei*	0.667	0.667	0.155
C3	* Lactobacillus rhamnosus *	0.836	0.568	0.887
C4	* Lactobacillus farciminis *	0.935	0.704	1.119
C5	* Lactobacillus bifermentans *	0.719	0.719	1.014

### Partial purification and molecular weight determination

**Fig. 1.**
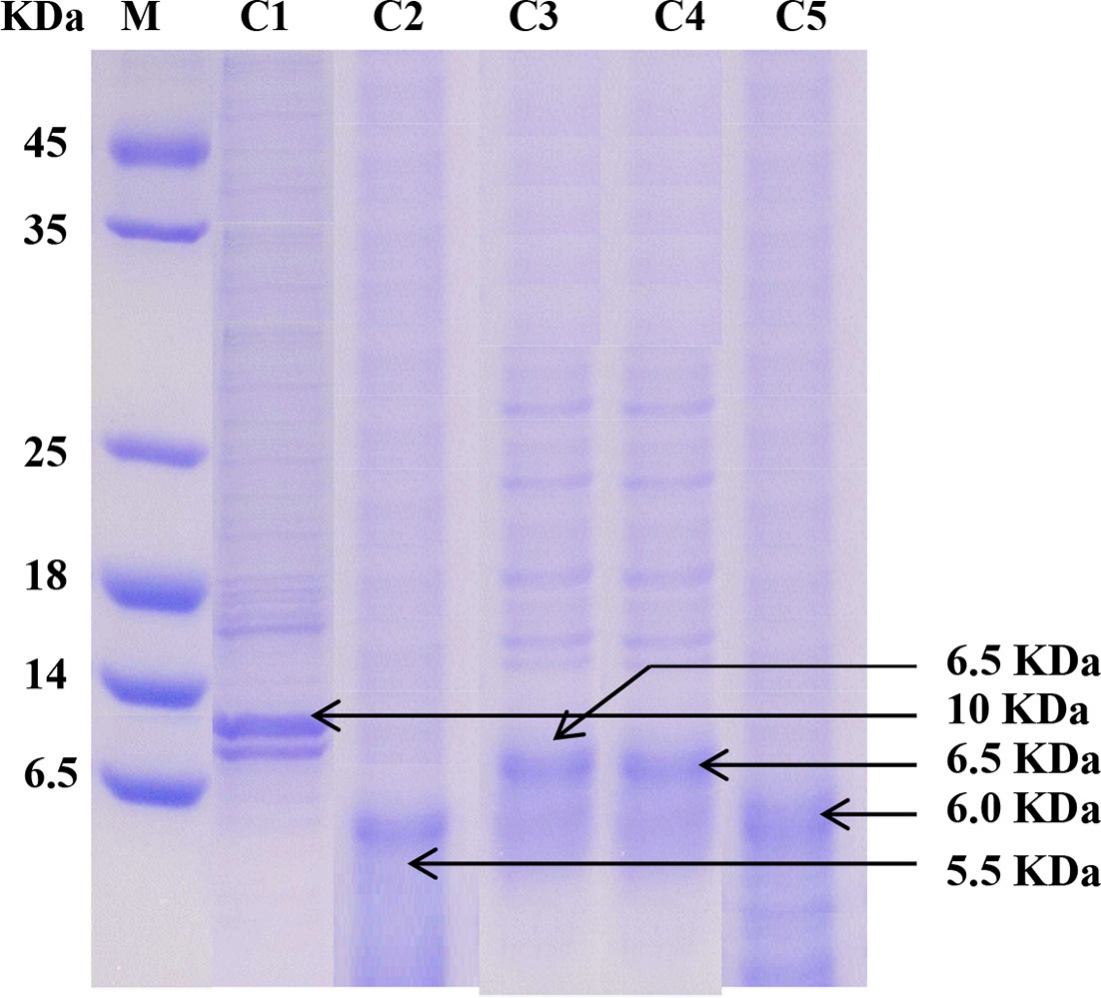
Molecular weight of extracted bacteriocins. Here, C1: *L. plantarum*, C2: *L. paracasei ss paracasei*, C3: *L. rhamnosus*, C4: *L. farciminis*, C5: *L. bifermentans* and M: molecular marker.

The extracted bacteriocin from *Lactobacillus* species were partially purified according to the modified method of [29, 28]. The bacteriocins appeared as distinct bands in SDS-PAGE with molecular weights around 7.0 to 10.5 kDa compared with the standard protein marker obtained from Promega, USA [17] (Fig. 1).

### Antimicrobial spectrum of bacteriocins

**Fig. 2.**
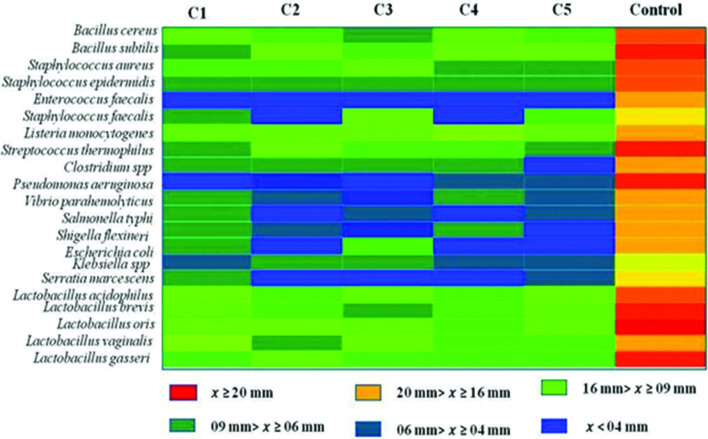
Inhibition zone map of extracted bacteriocins against indicators strains. Here, C1: *L. plantarum*, C2 : *L. paracasei* ss *paracasei*, C3: *L. rhamnosus*, C4: *L. farciminis*, C5: *L. bifermentans*, *x*: antimicrobial activity and ciprofloxacin used as a positive control.

A wide range of Gram-positive and Gram-negative bacteria were used to check the inhibition spectrum of these extracted bacteriocins and ciprofloxacin was used as a positive control. Different bacteriocins exhibited different inhibition profiles. Results are shown in Fig. 2. The obtained results indicated that most of the bacteriocins produced by LAB isolates are active against LABs and Gram-positive bacteria whereas the Gram-negative bacteria displayed considerable resistance. The extracted bacteriocins from all LAB isolates from cheese showed anti-*Listeria* activity are consistent with a previous report on bacteriocin-producing LAB isolates [6, 7, 22]. The bacteriocin of *L. paracasei* sub-species *paracasei* and *L. farciminis* strains were not antagonistic towards *S. typhi*, *S. flexineri* and *E. coli*. The bacteriocins from *L. rhamnosus* and *L. bifermentans* have very strong activity against *Bacillus* sp. and *Staphylococcus* spp. as well as enabling to inhibit *S. flexineri*, *E. coli* and *Klebsiella* spp. By contrast, a higher range of micro-organisms were inhibited at by the bacteriocin from *L. plantarum*isolates compared to bacteriocins from other strains. Previous results indicated that *L. plantarum* exhibits inhibitory activity against a wide of micro-organisms [39].

### Bacteriocin profiling and characterization

**Fig. 3.**
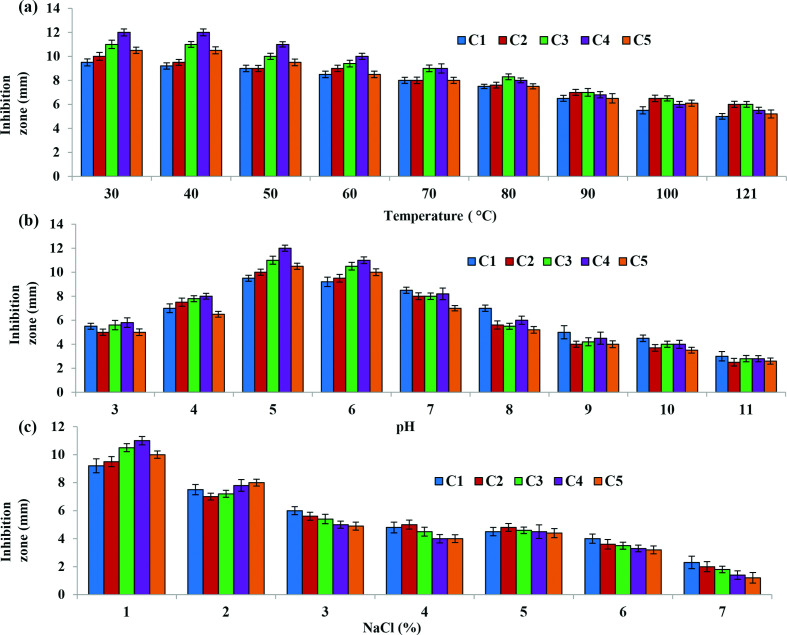
Effect of temperature, pH and NaCl concentrations on the antagonistic activity of extracted bacteriocins against *L. monocytogenes*. Here, C1: *L. plantarum*, C2: *L. paracasei ss paracasei*, C3: *L. rhamnosus*, C4: *L. farciminis*, C5: *L. bifermentans*.

Numerous studies have suggested that different environmental conditions such as temperature and pH can alter the activity of bacteriocins, thus the sensitivity of the bacteriocins to different physical conditions and chemical substances was evaluated. As, all the extracted bacteriocins in this study exhibited strong antimicrobial activity against *L. monocytogenes*, the stability of bacteriocins against *L. monocytogenes* is shown in Fig. 3. The stability of the bacteriocins against other pathogenic micro-organisms is given in Table S1(a–e), S2 (a–e) and S3 (a–e) (available in the online version of this article).

### Determination of MIC and MBC value

MIC and MBC value of bacteriocins were determined against *L. monocytogenes*. Table 2 shows the MIC and MBC value of bacteriocin against *L. monocytogenes*. The present findings are consistent with the previous report of bacteriocin from *Lactobacillius* spp. [40].

**Table 2.** MIC and MBC value of extracted bacteriocins against *L. monocytogenes*. (+) means growth appears, (-) means no growth

Test organism	Bacterial growth in Muller–Hinton medium (Bacteriocins concentrations)							
	50	100	200	300	400	500	MIC (mg ml^−1^)	MBC (mg ml^−1^)
**C1**	+	+	−	−	−	−	200	300
**C2**	+	+	−	−	−	−	200	300
**C3**	+	+	+	−	−	−	300	400
**C4**	+	+	−	−	−	−	200	300
**C5**	+	+	+	−	−	−	300	400

MBC, minimum bactericidal concentration; mg, milligram; MIC, minimum inhibitory concentration; ml, millilitre.

### Effect of temperature

Fig. 3a indicates the effect of temperature on bacteriocins activities against *L. monocytogenes,* in terms of inhibition zones. These results suggest the bacteriocins as thermostable in nature (Table S1).

### Effect of pH

Stability of the bacteriocins at diverse pH level (3–11) is a limiting factor for its use in food stuff, so the activity of extracted bacteriocin at different pH was estimated in this study. The result indicated that bacteriocins were active in a wide range of pH, and the greatest activity was observed at pH 4.0 to 6.0. However, bacteriocin could retain their antimicrobial activity partially when there was a shift to acidic or basic range (Fig. 3b, Table S2).

### Effect of NaCl concentrations

The effects of NaCl concentrations (1–7 %) on the activities of the five bacteriocins were studied. In almost all strains, 1.0 % NaCl increased the activities of bacteriocins. The bacteriocins from *L. rhamnosus* and *L. plantarum* dramatically decreased sensitivity in presence of 3.0 % NaCl concentrations but both showed the best sensitivity at 4.0 % NaCl concentrations (Fig. 3c, Table S3).

### Effect of enzymes and organic solvents

The effects of a number of enzymes (protease K, trypsin and amylase) on the activities of bacteriocins were assessed. The results demonstrated that the antimicrobial activities were decreased after treatment with the proteolytic enzymes, but were insensitive to amylase. The effects of organic solvents on bacteriocin activities were also evaluated (Table 3). The organic solvents were used as 1 : 1 ratio with bacteriocins. The results indicate that the bacteriocins were moderately sensitive to ethanol, methanol and acetone. n-butanol did not significantly affect the inhibitory activities of any bacteriocins.

**Table 3.** Antimicrobial activity of bacteriocins (treated with different enzymes and organic solvents) against *L. monocytogenes* (inhibition zones were measured as mm±sd)

Enzymes and organic solvents	Bacteriocin producing *Lactobacillus* sp*.*				
	C1(mm)	C2 (mm)	C3 (mm)	C4 (mm)	C5 (mm)
Protease K	5.5±0.5	6.0±0.4	6.5±0.5	7.0±0.4	7.5±0.5
Trypsin	6.5±0.4	7.0±0.7	7.5±0.4	5.5±0.3	6.5±0.6
Amylase	9.0±0.7	9.0±0.3	9.5±0.7	10.0±0.5	8.5±0.4
Ethanol	7.0±0.6	6.0±0.7	6.5±0.4	7.0±0.3	8.0±0.6
Methanol	8.5±0.6	7.5±0.4	7.0±0.6	8.0±0.7	7.5±0.5
Acetone	7.5±0.3	7.0±0.6	8.0±0.4	9.0±0.5	8.5±0.6
n-butanol	8.5±0.4	9.5±0.3	9.0±0.5	10.0±0.4	8.0±0.3
Control	9.5±0.7	10.0±0.6	11.0±0.3	12.0±0.4	10.5±0.5

*mm: millimetre.

SD, standard deviation.

### Effect of UV light

The antimicrobial activities of bacteriocins from LAB isolates were not affected by UV-light exposure within different time periods.

### Stability of bacteriocin during storage

Bacteriocins produced by different LAB isolates remained stable at −20 °C storage after 30 days. The activities of bacteriocins partially declined at 4 °C after storage for 60 days. In the case of optimum growth temperature (37 °C) no activity was detected after storage for 60 to 80 days indicating that relatively low temperature may be the most suitable technique for bacteriocins preservation.

## Discussion

A diverse number of bacterial microflora were found in different cheese samples, and numerous LAB isolates were also observed. Based on distinct morphological and physiological characteristics a total of 25 colonies were randomly picked from the tested samples. Finally, five isolates were properly identified up to species level through the BIOLOG system, specifically *L. plantarum*, *L. paracasei* subsp. paracasei*, L. rhamnosus, L. farciminis* and *L. bifermentans* selected for further study. In the current study, bacteriocins synthesized from lactobacillus species were partially purified by ammonium sulphate precipitation method [4, 28]. On the basis of SDS-PAGE analyses, the potential molecular weights of bacteriocins were determined to be approximately 7.0 to 10.5 kDa. The banding patterns were the same for all isolates that also suggests the isolates to belong to the identical genus. In a previous study, Todorov *et al*. [41] observed bacteriocin from *L. plantarum* ST13BR at 10.0 kDa regions. Similarly, Tolinacki *et al*. [42] also reported the bacteriocin from *L. paracasei* subsp. *paracasei* BGUB9 as 4.0 kDa. Moreover, the work of Dimitrijević *et al*. [43] indicated that the molecular weight of *L. rhamnosus* belonging to 6.5 kDa [43]. The bacteriocins from LAB isolates belonging to class-I and II have molecular weight (<5 kDa) and (<10 kDa), respectively [28, 43, 44]. Herein, the lower molecular weight of potential bacteriocins of LAB isolates implied that these belong to class-II bacteriocin group [17].

A wide range of bacterial pathogens were used to check the inhibition spectrum. The results indicate that the extracted bacteriocins have superior antimicrobial activity against *S. aureus*, *E. fecalis*, *E. coli* and *L. monocytogenes*. Similarly, bacteriocin from *L. plantarum* was found to be active against pathogenic bacteria including *Clostridium* sp*., E. fecalis, E. coli* and *S. aureus* [7, 39, 41]. Most of the bacteriocins produced by LAB are active only against LAB and other Gram-positive bacteria. Currently, outbreaks of listeriosis are a serious food-borne disease that attract public attention and are of concern to those responsible for food safety [45, 46]. All the bacteriocins extracted in this work inhibited markedly the growth of *L. monocytogenes*. Similar results were obtained by Pitt *et al.* [40] having used milk that was experimentally contaminated with *Listeria* cultures in the presence and in the absence of pediocin 5. Tests were made on raw milk contaminated artificially by *L. monocytogenes* in both the presence and absence of bacteriocin produced by *Carnobacterium piscicola* JG 126. Under these conditions, piscicolin 126 reduced the *Listeria* species from 4 to 5 log during storage [40].

The activities of extracted bacteriocins from all these LAB isolates were stable within a broad range of temperatures at 40–70 °C for 20 min (Fig. 3a). Following heat treatment at 121 °C for 20 min, bacteriocins retained around 90 % of its original antibacterial activity. In contrast, the activity of bacteriocins remains for 2 months at 4 °C, and 3 to 4 months at −20 °C. Thermostability of bacteriocins at both high and low temperature makes it possible to sterilize the food products even at room temperature, and can thus be applied in food-related industries. The antimicrobial actions of all bacteriocins were constant at pH 4.0 to 8.0 (Fig. 3b). A previous study demonstrated that nisin shows an excellent antimicrobial affinity at lower pH value and inactivated at pH close to 7.0 [19, 47]. In the case of NaCl, the antimicrobial activities of bacteriocins remain at 1.0–3.0 % of NaCl application (Fig. 3c).

The enzymatic reaction of bacteriocins from all LAB isolates exhibit that the bacteriocins were degraded by trypsin and protease K as well as moderately responsive to amylase, signifying the peptide feature of the bacteriocins. The previous observations reported that bacteriocins have unusual sensitivities to different enzymes, such as mesenterocin E131 entirely inactivated by trypsin and proteinase K as well as *Leuconostoc paramesenteroides* are sensitive to amylase but resistant to protease [48]. These findings suggest that the bacteriocins are mostly protein in nature and therefore can be broken down by gastric juices, and as a result it can be used for human consumption [49].

In conclusion, the LAB bacteriocins from cheese samples were efficient in inhibiting pathogenic organisms. The broad inhibitory spectrum of the bacteriocins substances suggests that they may have a potential application as bio-preservatives in food or related industries.

### Data availability statement

The experimental data used to support the findings of this study are available from the corresponding author upon request.

## Supplementary Data

Supplementary material 1
