# Supplementary material for: Characterization and profiling of bacteriocin-like substances produced by lactic acid bacteria from cheese samples
Source: Access Microbiol. 2021 Jun 8;3(6):000234. doi: 10.1099/acmi.0.000234 (PMC8374546; doi:10.1099/acmi.0.000234)
Supplement: Supplementary material 1 [file acmi-3-0234-s001.pdf]

## Supplementary material

**01a.** Effect of temperature on the antagonistic activity of extracted bacteriocins of *L. plantarum* against different bacterial pathogens. Inhibition zone were measured as mm ± Standard deviation.

| **Indicator strains** | **Bacteriocin producing *L. plantarum*** | | | | | | | | |
|---|---|---|---|---|---|---|---|---|---|
| | **30**°C | **40**°C | **50**°C | **60**°C | **70**°C | **80**°C | **90**°C | **100**°C | **121**°C |
| *Bacillus cereus* | 9.0± 0.5 | 8.4 ± 0.9 | 7.6 ± 0.3 | 6.5 ± 0.3 | 6.0 ± 0.5 | 5.5 ± 0.8 | 5.0 ± 0.9 | 5.0 ± 0.7 | 4.0 ± 0.4 |
| *Bacillus subtilis* | 7.0 ± 0.3 | 7.0 ± 0.5 | 6.5 ± 0.3 | 6.3 ± 0.4 | 6.0 ± 0.3 | 6.0 ± 0.5 | 4.0 ± 0.3 | 4.2 ± 0.6 | 3.0 ± 0.3 |
| *Staphylococcus aureus* | 8.0 ± 0.3 | 7.5 ± 0.4 | 7.5 ± 0.5 | 6.0 ± 0.5 | 5.0 ± 0.6 | 5.0 ± 0.3 | 4.5 ± 0.7 | 4.0 ± 0.3 | 2.5 ± 0.3 |
| *Staphylococcus epidermidis* | 7.0 ± 0.6 | 6.0 ± 0.3 | 6.0 ± 0.5 | 5.8 ± 0.3 | 5.3 ± 0.5 | 5.0 ± 0.3 | 4.3 ± 0.5 | 3.3 ± 0.4 | 3.0 ± 0.5 |
| *Enterococcus faecalis* | - | - | - | - | - | - | - | - | - |
| *Staphylococcus faecalis* | 6.5 ± 0.5 | 6.0 ± 0.7 | 5.5 ± 0.6 | 4.5 ± 0.5 | 4.5 ± 0.3 | 4.2 ± 0.5 | 4.0 ± 0.4 | 4.0 ± 0.5 | 3.5 ± 0.7 |
| *Listeria monocytogenes* | 9.5 ± 0.7 | 9.2 ± 0.6 | 9.0 ± 0.3 | 8.5 ± 0.4 | 8.0 ± 0.5 | 7.5 ± 0.6 | 6.5 ± 0.3 | 5.5 ± 0.4 | 5.0 ± 0.4 |
| *Streptococcus thermophilus* | 6.0 ± 0.5 | 6.0 ± 0.6 | 5.3 ± 0.3 | 4.5 ± 0.5 | 4.5 ± 0.4 | 4.0 ± 0.7 | 3.2 ± 0.8 | 3.0 ± 0.5 | 2.4 ± 0.5 |
| *Clostridium spp.* | 5.5 ± 0.4 | 5.3 ± 0.5 | 4.8 ± 0.3 | 4.4 ± 0.4 | 4.0 ± 0.5 | 4.0 ± 0.4 | 3.0 ± 0.9 | 2.5 ± 0.8 | 2.5 ± 0.4 |
| *Pseudomonas aeruginosa* | - | - | - | - | - | - | - | - | - |
| *Vibrio parahemolyticus* | 6.5 ± 0.5 | 5.5 ± 0.3 | 5.5 ± 0.5 | 5.2 ± 0.3 | 4.0 ± 0.6 | 4.0 ± 0.8 | 3.3 ± 0.4 | 3.0 ± 0.9 | 3.0 ± 0.6 |
| *Salmonella typhi* | 5.5 ± 0.3 | 5.5 ± 0.7 | 5.3 ± 0.3 | 5.0 ± 0.3 | 4.0 ± 0.3 | 3.5 ± 0.5 | 3.5 ± 0.6 | 2.5 ± 0.3 | 2.2 ± 0.7 |
| *Shigella flexineri* | 4.5 ± 0.3 | 4.2 ± 0.5 | 4.0 ± 0.8 | 4.0 ± 0.9 | 3.5 ± 0.6 | 3.3 ± 0.4 | 3.3 ± 0.5 | 2.5 ± 0.5 | 2.0 ± 0.9 |
| *Escherichia coli* | 6.0 ± 0.5 | 6.0 ± 0.9 | 5.5 ± 0.5 | 5.4 ± 0.3 | 5.0 ± 0.5 | 5.0 ± 0.6 | 4.4 ± 0.5 | 4.0 ± 0.3 | 3.2 ± 0.5 |
| *Klebsiella spp.* | 5.0 ± 0.5 | 4.5 ± 0.3 | 4.2 ± 0.3 | 4.0 ± 0.5 | 4.0 ± 0.3 | 3.2 ± 0.6 | 3.0 ± 0.4 | 3.0 ± 0.9 | 2.3 ± 0.3 |
| *Serratia marcescens* | 6.5 ± 0.5 | 6.2 ± 0.9 | 6.2 ± 0.7 | 6.0 ± 0.5 | 5.3 ± 0.6 | 4.5 ± 0.8 | 4.5 ± 0.5 | 3.5 ± 0.5 | 3.0 ±0.4 |
| *Lactobacillus acidophilus* | 9.5 ± 0.5 | 8.5 ± 0.8 | 8.3 ± 0.5 | 8.0 ± 0.5 | 7.5 ± 0.6 | 7.5 ± 0.5 | 6.0 ± 0.9 | 5.5 ± 0.5 | 4.0 ± 0.7 |
| *Lactobacillus brevis* | 9.5 ± 0.3 | 7.5 ± 0.5 | 6.0 ± 0.4 | 6.0 ± 0.3 | 5.0 ± 0.8 | 4.0 ± 0.5 | 4.0 ± 0.7 | 3.3 ± 0.6 | 3.0 ± 0.5 |
| *Lactobacillus oris* | 8.5 ± 0.4 | 7.0 ± 0.3 | 6.5 ± 0.5 | 6.0 ± 0.5 | 6.0 ± 0.9 | 5.0 ± 0.3 | 4.3 ± 0.7 | 4.0 ± 0.8 | 3.7 ± 0.9 |
| *Lactobacillus vaginalis* | 10.5 ± 0.5 | 9.0 ± 0.5 | 9.0 ± 0.4 | 9.0 ± 0.3 | 8.5 ± 0.6 | 7.3 ± 0.9 | 7.0 ± 0.8 | 7.0 ± 0.6 | 5.5 ± 0.5 |
| *Lactobacillus gasseri* | 8.0 ± 0.5 | 8.0 ± 0.5 | 7.5 ± 0.5 | 6.3 ± 0.3 | 6.0 ± 0.4 | 6.0 ± 0.6 | 5.4 ± 0.4 | 5.0 ± 0.7 | 4.3 ± 0.8 |

**01b.** Effect of temperature on the antagonistic activity of extracted bacteriocins of *L. paracasei ss paracasei* against different bacterial pathogens. Inhibition zone were measured as mm ± Standard deviation.

| **Indicator strains** | **Bacteriocin producing *L. paracasei ss paracasei*** | | | | | | | | |
|---|---|---|---|---|---|---|---|---|---|
| | **30**°C | **40**°C | **50**°C | **60**°C | **70**°C | **80**°C | **90**°C | **100**°C | **121**°C |
| *Bacillus cereus* | 8.0 ± 0.3 | 8.0 ± 0.5 | 7.4 ± 0.9 | 6.2 ± 0.7 | 6.0 ± 0.5 | 6.0 ± 0.8 | 5.5 ± 0.9 | 5.0 ± 0.6 | 3.0 ± 0.4 |
| *Bacillus subtilis* | 7.8 ± 0.6 | 7.5 ± 0.7 | 7.0 ± 0.4 | 6.0 ± 0.3 | 6.0 ± 0.5 | 5.0 ± 0.5 | 4.5 ± 0.3 | 4.2 ± 0.7 | 2.5 ± 0.9 |
| *Staphylococcus aureus* | 8.4 ± 0.7 | 7.2 ± 0.6 | 7.0 ± 0.5 | 6.5 ± 0.4 | 6.0 ± 0.3 | 5.5 ± 0.4 | 4.0 ± 0.3 | 3.3 ± 0.4 | 2.2 ± 0.5 |
| *Staphylococcus epidermidis* | 7.8 ± 0.6 | 6.8 ± 0.5 | 6.0 ± 0.3 | 5.5 ± 0.6 | 5.0 ± 0.5 | 4.0 ± 0.7 | 3.5 ± 0.4 | 3.0 ± 0.5 | 3.0 ± 0.9 |
| *Enterococcus faecalis* | - | - | - | - | - | - | - | - | - |
| *Staphylococcus faecalis* | 7.8 ± 0.6 | 7.0 ± 0.5 | 6.4 ± 0.3 | 5.5 ± 0.5 | 5.5 ± 0.5 | 5.2 ± 0.7 | 4.0 ± 0.5 | 4.0 ± 0.5 | 2.5 ± 0.4 |
| *Listeria monocytogenes* | 10.0 ± 0.7 | 9.5 ± 0.6 | 9.0 ± 0.3 | 9.0 ± 0.4 | 8.0 ± 0.5 | 7.6 ± 0.6 | 7.0 ± 0.3 | 6.5 ± 0.4 | 6.0 ± 0.4 |
| *Streptococcus thermophilus* | 7.0 ± 0.6 | 6.0 ± 0.7 | 5.1 ± 0.3 | 4.6 ± 0.5 | 4.5 ± 0.4 | 4.0 ± 0.3 | 3.3 ± 0.8 | 3.0 ± 0.5 | 2.2 ± 0.3 |
| *Clostridium spp.* | 6.5 ± 0.5 | 6.0 ± 0.4 | 5.8 ± 0.3 | 5.4 ± 0.4 | 5.0 ± 0.7 | 4.6 ± 0.4 | 4.0 ± 0.9 | 3.5 ± 0.8 | 2.0 ± 0.3 |
| *Pseudomonas aeruginosa* | - | - | - | - | - | - | - | - | - |
| *Vibrio parahemolyticus* | 6.0 ± 0.3 | 5.0 ± 0.4 | 5.0 ± 0.5 | 4.2 ± 0.7 | 4.2 ± 0.6 | 4.0 ± 0.8 | 3.5 ± 0.4 | 3.2 ± 0.9 | 3.0 ± 0.6 |
| *Salmonella typhi* | 6.5 ± 0.5 | 6.2 ± 0.7 | 5.8 ± 0.4 | 5.6 ± 0.3 | 5.0 ± 0.4 | 4.5 ± 0.5 | 3.5 ± 0.6 | 2.0 ± 0.6 | 2.0 ± 0.7 |
| *Shigella flexineri* | 5.5 ± 0.3 | 4.2 ± 0.5 | 3.8 ± 0.8 | 3.8 ± 0.9 | 3.5 ± 0.6 | 3.0 ± 0.4 | 3.0 ± 0.5 | 2.8 ± 0.5 | 2.5 ± 0.9 |
| *Escherichia coli* | 6.5 ± 0.5 | 6.0 ± 0.6 | 5.0 ± 0.5 | 5.1 ± 0.8 | 5.1 ± 0.5 | 4.5 ± 0.6 | 4.4 ± 0.7 | 4.0 ± 0.3 | 3.0 ± 0.5 |
| *Klebsiella spp.* | 6.0 ± 0.5 | 5.5 ± 0.3 | 5.0 ± 0.3 | 5.0 ± 0.5 | 4.5 ± 0.3 | 3.0 ± 0.6 | 3.0 ± 0.4 | 3.0 ± 0.5 | 2.0 ± 0.4 |
| *Serratia marcescens* | 6.2 ± 0.5 | 6.0 ± 0.6 | 6.0 ± 0.7 | 5.0 ± 0.9 | 5.3 ± 0.6 | 4.8 ± 0.8 | 4.0 ± 0.5 | 3.5 ± 0.5 | 3.0 ±0.3 |
| *Lactobacillus acidophilus* | 9.0 ± 0.7 | 8.5 ± 0.8 | 8.5 ± 0.5 | 8.0 ± 0.3 | 7.5 ± 0.6 | 7.0 ± 0.5 | 6.0 ± 0.9 | 5.5 ± 0.5 | 3.5 ± 0.7 |
| *Lactobacillus brevis* | 9.5 ± 0.4 | 8.5 ± 0.5 | 8.0 ± 0.4 | 7.0 ± 0.3 | 6.0 ± 0.8 | 6.0 ± 0.5 | 4.8 ± 0.7 | 3.0 ± 0.6 | 3.0 ± 0.5 |
| *Lactobacillus oris* | 8.0 ± 0.3 | 7.5 ± 0.3 | 6.8 ± 0.5 | 6.0 ± 0.5 | 6.0 ± 0.9 | 5.5 ± 0.3 | 4.0 ± 0.7 | 3.2 ± 0.8 | 3.2 ± 0.9 |
| *Lactobacillus vaginalis* | 10.0 ± 0.6 | 9.5 ± 0.3 | 9.0 ± 0.5 | 9.0 ± 0.6 | 8.0 ± 0.3 | 7.0 ± 0.9 | 6.5 ± 0.8 | 6.5 ± 0.6 | 4.5 ± 0.5 |
| *Lactobacillus gasseri* | 9.0 ± 0.7 | 8.5 ± 0.5 | 7.0 ± 0.3 | 6.5 ± 0.5 | 6.0 ± 0.7 | 6.0 ± 0.6 | 5.5 ± 0.6 | 5.0 ± 0.7 | 4.0 ± 0.8 |

**01c.** Effect of temperature on the antagonistic activity of extracted bacteriocins of *L. rhamnosus* against different bacterial pathogens. Inhibition zone were measured as mm ± Standard deviation.

| **Indicator strains** | **Bacteriocin producing *L. rhamnosus*** | | | | | | | | |
|---|---|---|---|---|---|---|---|---|---|
| | **30**°C | **40**°C | **50**°C | **60**°C | **70**°C | **80**°C | **90**°C | **100**°C | **121**°C |
| *Bacillus cereus* | 8.5 ± 0.4 | 8.0 ± 0.5 | 7.5 ± 0.3 | 6.0 ± 0.4 | 6.0 ± 0.5 | 5.2 ± 0.5 | 5.0 ± 0.9 | 5.0 ± 0.7 | 3.0 ± 0.4 |
| *Bacillus subtilis* | 6.5 ± 0.5 | 6.0 ± 0.3 | 6.0 ± 0.3 | 5.3 ± 0.5 | 5.0 ± 0.3 | 5.0 ± 0.3 | 4.6 ± 0.5 | 4.0 ± 0.6 | 3.2 ± 0.3 |
| *Staphylococcus aureus* | 7.0 ± 0.3 | 7.0 ± 0.4 | 6.5 ± 0.5 | 6.0 ± 0.6 | 6.0 ± 0.3 | 5.2 ± 0.5 | 4.3 ± 0.5 | 4.0 ± 0.7 | 2.0 ± 0.3 |
| *Staphylococcus epidermidis* | 7.0 ± 0.6 | 7.0 ± 0.3 | 6.2 ± 0.5 | 5.5 ± 0.3 | 5.2 ± 0.5 | 5.2 ± 0.5 | 4.0 ± 0.6 | 3.0 ± 0.7 | 3.0 ± 0.8 |
| *Enterococcus faecalis* | - | - | - | - | - | - | - | - | - |
| *Staphylococcus faecalis* | 6.0 ± 0.6 | 5.0 ± 0.5 | 5.0 ± 0.3 | 4.8 ± 0.4 | 4.5 ± 0.4 | 4.2 ± 0.3 | 4.0 ± 0.5 | 4.0 ± 0.3 | 3.2 ± 0.3 |
| *Listeria monocytogenes* | 11.0 ± 0.7 | 11.0 ± 0.6 | 10.0 ± 0.3 | 9.4 ± 0.4 | 9.0 ± 0.5 | 8.0 ± 0.6 | 7.0 ± 0.3 | 6.5 ± 0.4 | 6.0 ± 0.4 |
| *Streptococcus thermophilus* | 6.5 ± 0.5 | 6.5 ± 0.6 | 5.5 ± 0.3 | 5.5 ± 0.5 | 4.5 ± 0.7 | 4.0 ± 0.8 | 3.3 ± 0.3 | 3.0 ± 0.4 | 2.0 ± 0.5 |
| *Clostridium spp.* | 5.0 ± 0.5 | 4.8 ± 0.4 | 4.5 ± 0.3 | 4.3 ± 0.6 | 4.0 ± 0.5 | 4.0 ± 0.9 | 3.2 ± 0.3 | 2.8 ± 0.7 | 2.5 ± 0.4 |
| *Pseudomonas aeruginosa* | - | - | - | - | - | - | - | - | - |
| *Vibrio parahemolyticus* | 6.0 ± 0.5 | 6.0 ± 0.3 | 5.5 ± 0.5 | 5.0 ± 0.3 | 4.2 ± 0.6 | 4.2 ± 0.8 | 3.3 ± 0.4 | 3.0 ± 0.9 | 3.0 ± 0.6 |
| *Salmonella typhi* | 5.5 ± 0.3 | 5.0 ± 0.7 | 5.0 ± 0.3 | 4.6 ± 0.3 | 4.0 ± 0.3 | 3.0 ± 0.5 | 3.0 ± 0.6 | 2.2 ± 0.3 | 2.2 ± 0.7 |
| *Shigella flexineri* | 4.5 ± 0.5 | 4.0 ± 0.6 | 4.0 ± 0.7 | 3.5 ± 0.9 | 3.5 ± 0.5 | 3.2 ± 0.3 | 3.0 ± 0.6 | 2.5 ± 0.5 | 2.5 ± 0.4 |
| *Escherichia coli* | 6.5 ± 0.6 | 6.0 ± 0.7 | 6.0 ± 0.4 | 5.5 ± 0.3 | 5.2 ± 0.3 | 5.0 ± 0.5 | 4.5 ± 0.7 | 4.0 ± 0.3 | 3.0 ± 0.5 |
| *Klebsiella spp.* | 5.5 ± 0.4 | 5.5 ± 0.3 | 5.2 ± 0.5 | 4.6 ± 0.5 | 4.0 ± 0.3 | 3.3 ± 0.6 | 3.2 ± 0.3 | 3.0 ± 0.4 | 2.0 ± 0.5 |
| *Serratia marcescens* | 6.0 ± 0.6 | 6.0 ± 0.5 | 5.4 ± 0.9 | 5.0 ± 0.5 | 5.0 ± 0.5 | 4.6 ± 0.4 | 4.5 ± 0.5 | 3.5 ± 0.3 | 2.5 ±0.4 |
| *Lactobacillus acidophilus* | 7.5 ± 0.5 | 7.5 ± 0.3 | 7.3 ± 0.4 | 7.0 ± 0.5 | 6.5 ± 0.4 | 6.5 ± 0.5 | 6.0 ± 0.5 | 5.5 ± 0.5 | 4.5 ± 0.3 |
| *Lactobacillus brevis* | 9.0 ± 0.5 | 8.5 ± 0.4 | 8.0 ± 0.3 | 8.0 ± 0.6 | 7.5 ± 0.7 | 7.5 ± 0.9 | 6.0 ± 0.7 | 5.3 ± 0.5 | 4.0 ± 0.5 |
| *Lactobacillus oris* | 8.0 ± 0.4 | 7.8 ± 0.4 | 7.5 ± 0.5 | 6.2 ± 0.7 | 6.0 ± 0.5 | 5.5 ± 0.3 | 4.0 ± 0.6 | 4.0 ± 0.5 | 3.5 ± 0.4 |
| *Lactobacillus vaginalis* | 10.0 ± 0.6 | 10.0 ± 0.5 | 9.0 ± 0.3 | 9.0 ± 0.4 | 7.5 ± 0.5 | 7.3 ± 0.7 | 6.0 ± 0.8 | 6.0 ± 0.4 | 5.2 ± 0.5 |
| *Lactobacillus gasseri* | 8.5 ± 0.7 | 8.0 ± 0.8 | 7.5 ± 0.5 | 6.3 ± 0.3 | 6.0 ± 0.3 | 6.0 ± 0.4 | 5.0 ± 0.4 | 5.0 ± 0.5 | 4.0 ± 0.6 |

**01d.** Effect of temperature on the antagonistic activity of extracted bacteriocins of C4: *L. farciminis* against different bacterial pathogens. Inhibition zone were measured as mm ± Standard deviation.

| **Indicator strains** | **Bacteriocin producing *L. farciminis*** | | | | | | | | |
|---|---|---|---|---|---|---|---|---|---|
| | **30**°C | **40**°C | **50**°C | **60**°C | **70**°C | **80**°C | **90**°C | **100**°C | **121**°C |
| *Bacillus cereus* | 9.0 ± 0.3 | 8.5 ± 0.4 | 7.6 ± 0.5 | 6.4 ± 0.7 | 6.0 ± 0.6 | 5.5 ± 0.8 | 4.5 ± 0.7 | 4.5 ± 0.6 | 3.0 ± 0.5 |
| *Bacillus subtilis* | 7.5 ± 0.5 | 7.0 ± 0.4 | 6.0 ± 0.3 | 6.0 ± 0.4 | 5.0 ± 0.5 | 5.0 ± 0.5 | 4.2 ± 0.3 | 4.2 ± 0.5 | 2.5 ± 0.4 |
| *Staphylococcus aureus* | 8.2 ± 0.5 | 7.6 ± 0.5 | 7.0 ± 0.4 | 6.0 ± 0.3 | 5.5 ± 0.3 | 5.0 ± 0.4 | 4.5 ± 0.5 | 4.0 ± 0.3 | 2.5 ± 0.3 |
| *Staphylococcus epidermidis* | 7.0 ± 0.4 | 6.5 ± 0.3 | 6.0 ± 0.7 | 5.5 ± 0.9 | 5.3 ± 0.6 | 5.0 ± 0.5 | 4.5 ± 0.5 | 3.0 ± 0.4 | 3.0 ± 0.3 |
| *Enterococcus faecalis* | - | - | - | - | - | - | - | - | - |
| *Staphylococcus faecalis* | 7.5 ± 0.7 | 6.0 ± 0.4 | 5.0 ± 0.8 | 5.0 ± 0.9 | 4.5 ± 0.5 | 4.2 ± 0.5 | 4.2 ± 0.7 | 4.0 ± 0.5 | 3.5 ± 0.4 |
| *Listeria monocytogenes* | 10.0 ± 0.6 | 10.0 ± 0.7 | 9.0 ± 0.3 | 8.0 ± 0.3 | 8.0 ± 0.4 | 7.2 ± 0.6 | 6.8 ± 0.5 | 6.0 ± 0.4 | 5.0 ± 0.3 |
| *Streptococcus thermophilus* | 6.0 ± 0.7 | 5.5 ± 0.3 | 5.0 ± 0.4 | 5.0 ± 0.5 | 4.8 ± 0.5 | 4.0 ± 0.6 | 3.0 ± 0.7 | 3.0 ± 0.5 | 2.5 ± 0.4 |
| *Clostridium spp.* | 6.5 ± 0.8 | 5.3 ± 0.5 | 4.8 ± 0.5 | 4.5 ± 0.4 | 3.5 ± 0.5 | 3.5 ± 0.4 | 3.0 ± 0.9 | 2.5 ± 0.8 | 2.5 ± 0.4 |
| *Pseudomonas aeruginosa* | - | - | - | - | - | - | - | - | - |
| *Vibrio parahemolyticus* | 5.5 ± 0.8 | 5.2 ± 0.4 | 5.0 ± 0.5 | 5.0 ± 0.7 | 4.5 ± 0.7 | 4.0 ± 0.5 | 3.5 ± 0.4 | 3.0 ± 0.8 | 2.8 ± 0.7 |
| *Salmonella typhi* | 6.5 ± 0.6 | 5.5 ± 0.8 | 5.0 ± 0.9 | 4.8 ± 0.5 | 4.0 ± 0.5 | 3.0 ± 0.3 | 3.0 ± 0.6 | 2.5 ± 0.3 | 2.5 ± 0.8 |
| *Shigella flexineri* | 5.5 ± 0.6 | 5.2 ± 0.7 | 5.0 ± 0.8 | 4.5 ± 0.5 | 4.0 ± 0.6 | 4.0 ± 0.5 | 3.5 ± 0.4 | 3.5 ± 0.4 | 3.0 ± 0.7 |
| *Escherichia coli* | 6.5 ± 0.5 | 6.5 ± 0.5 | 5.5 ± 0.8 | 5.4 ± 0.9 | 5.0 ± 0.6 | 5.0 ± 0.6 | 4.5 ± 0.5 | 4.0 ± 0.3 | 3.3 ± 0.5 |
| *Klebsiella spp.* | 5.5 ± 0.5 | 4.5 ± 0.3 | 4.0 ± 0.3 | 4.0 ± 0.5 | 3.5 ± 0.3 | 3.5 ± 0.6 | 3.0 ± 0.4 | 3.0 ± 0.9 | 2.5 ± 0.3 |
| *Serratia marcescens* | 6.0 ± 0.7 | 6.0 ± 0.5 | 5.5 ± 0.7 | 5.0 ± 0.8 | 5.0 ± 0.6 | 4.8 ± 0.6 | 4.5 ± 0.5 | 3.5 ± 0.4 | 2.5 ±0.3 |
| *Lactobacillus acidophilus* | 9.0 ± 0.6 | 8.0 ± 0.7 | 8.0 ± 0.8 | 7.8 ± 0.4 | 7.8 ± 0.6 | 7.0 ± 0.6 | 6.0 ± 0.8 | 5.5 ± 0.6 | 3.0 ± 0.4 |
| *Lactobacillus brevis* | 9.0 ± 0.5 | 7.5 ± 0.3 | 7.0 ± 0.6 | 6.0 ± 0.4 | 5.0 ± 0.7 | 4.0 ± 0.7 | 4.0 ± 0.7 | 3.0 ± 0.4 | 3.0 ± 0.5 |
| *Lactobacillus oris* | 8.0 ± 0.6 | 7.8 ± 0.4 | 6.8 ± 0.7 | 6.5 ± 0.5 | 6.0 ± 0.4 | 5.5 ± 0.3 | 4.0 ± 0.8 | 4.0 ± 0.5 | 3.5 ± 0.7 |
| *Lactobacillus vaginalis* | 10.0 ± 0.6 | 10.0 ± 0.8 | 9.6 ± 0.4 | 9.0 ± 0.5 | 8.6 ± 0.7 | 6.5 ± 0.4 | 6.5 ± 0.8 | 6.0 ± 0.7 | 5.5 ± 0.5 |
| *Lactobacillus gasseri* | 8.5 ± 0.6 | 8.5 ± 0.5 | 7.0 ± 0.3 | 6.5 ± 0.3 | 6.0 ± 0.4 | 6.0 ± 0.4 | 5.5 ± 0.6 | 5.0 ± 0.7 | 4.0 ± 0.5 |

**01e.** Effect of temperature on the antagonistic activity of extracted bacteriocins of *L. bifermentans* against different bacterial pathogens. Inhibition zone were measured as mm ± standard deviation.

| **Indicator strains** | **Bacteriocin producing *L. bifermentans*** | | | | | | | | |
|---|---|---|---|---|---|---|---|---|---|
| | **30**°C | **40**°C | **50**°C | **60**°C | **70**°C | **80**°C | **90**°C | **100**°C | **121**°C |
| *Bacillus cereus* | 8.4 ± 0.6 | 8.0 ± 0.4 | 7.5 ± 0.3 | 6.8 ± 0.5 | 5.5 ± 0.6 | 5.5 ± 0.9 | 5.0 ± 0.6 | 4.0 ± 0.7 | 3.0 ± 0.3 |
| *Bacillus subtilis* | 6.0 ± 0.4 | 6.0 ± 0.5 | 5.5 ± 0.3 | 5.3 ± 0.3 | 5.0 ± 0.4 | 4.0 ± 0.3 | 4.0 ± 0.5 | 3.2 ± 0.5 | 3.0 ± 0.6 |
| *Staphylococcus aureus* | 7.0 ± 0.5 | 6.8 ± 0.4 | 6.5 ± 0.3 | 6.0 ± 0.3 | 5.0 ± 0.5 | 5.0 ± 0.5 | 4.5 ± 0.6 | 4.0 ± 0.3 | 2.5 ± 0.7 |
| *Staphylococcus epidermidis* | 6.5 ± 0.4 | 6.0 ± 0.6 | 6.0 ± 0.5 | 5.5 ± 0.7 | 5.0 ± 0.9 | 5.0 ± 0.5 | 4.0 ± 0.3 | 3.0 ± 0.5 | 3.0 ± 0.3 |
| *Enterococcus faecalis* | - | - | - | - | - | - | - | - | - |
| *Staphylococcus faecalis* | 5.5 ± 0.3 | 5.0 ± 0.5 | 5.0 ± 0.3 | 4.8 ± 0.4 | 4.5 ± 0.5 | 4.5 ± 0.5 | 4.0 ± 0.3 | 4.0 ± 0.5 | 3.0 ± 0.4 |
| *Listeria monocytogenes* | 10.5 ± 0.7 | 10.5 ± 0.6 | 9.5 ± 0.3 | 8.5 ± 0.4 | 8.0 ± 0.5 | 7.5 ± 0.6 | 6.5 ± 0.3 | 6.1 ± 0.4 | 5.2 ± 0.4 |
| *Streptococcus thermophilus* | 5.0 ± 0.6 | 5.0 ± 0.7 | 4.5 ± 0.8 | 4.5 ± 0.9 | 4.0 ± 0.4 | 4.0 ± 0.3 | 3.5 ± 0.7 | 3.0 ± 0.5 | 2.5 ± 0.3 |
| *Clostridium spp.* | 5.5 ± 0.7 | 5.0 ± 0.5 | 4.8 ± 0.3 | 4.6 ± 0.5 | 4.5 ± 0.4 | 4.0 ± 0.7 | 3.5 ± 0.4 | 2.5 ± 0.4 | 2.0 ± 0.6 |
| *Pseudomonas aeruginosa* | - | - | - | - | - | - | - | - | - |
| *Vibrio parahemolyticus* | 6.0 ± 0.8 | 6.0 ± 0.7 | 5.5 ± 0.5 | 5.5 ± 0.6 | 4.0 ± 0.3 | 4.0 ± 0.5 | 3.5 ± 0.4 | 3.0 ± 0.3 | 2.5 ± 0.5 |
| *Salmonella typhi* | 5.0 ± 0.4 | 5.0 ± 0.5 | 4.8 ± 0.3 | 4.8 ± 0.3 | 4.5 ± 0.5 | 4.5 ± 0.6 | 3.5 ± 0.7 | 2.8 ± 0.4 | 2.5 ± 0.7 |
| *Shigella flexineri* | 4.5 ± 0.5 | 4.5 ± 0.3 | 4.0 ± 0.7 | 4.0 ± 0.4 | 3.0 ± 0.3 | 3.0 ± 0.6 | 2.5 ± 0.5 | 2.5 ± 0.4 | 2.0 ± 0.9 |
| *Escherichia coli* | 6.0 ± 0.3 | 6.0 ± 0.4 | 5.5 ± 0.7 | 5.5 ± 0.6 | 5.0 ± 0.5 | 5.0 ± 0.7 | 4.5 ± 0.5 | 4.0 ± 0.8 | 3.5 ± 0.5 |
| *Klebsiella spp.* | 4.5 ± 0.4 | 4.5 ± 0.3 | 4.0 ± 0.5 | 4.0 ± 0.3 | 3.5 ± 0.4 | 3.5 ± 0.4 | 3.0 ± 0.6 | 3.0 ± 0.7 | 2.5 ± 0.8 |
| *Serratia marcescens* | 6.5 ± 0.5 | 6.2 ± 0.6 | 6.2 ± 0.5 | 6.0 ± 0.7 | 5.3 ± 0.5 | 4.5 ± 0.8 | 4.5 ± 0.6 | 3.5 ± 0.5 | 3.0 ±0.7 |
| *Lactobacillus acidophilus* | 9.0 ± 0.4 | 8.5 ± 0.6 | 8.5 ± 0.7 | 8.0 ± 0.5 | 7.5 ± 0.8 | 7.5 ± 0.7 | 7.0 ± 0.6 | 6.5 ± 0.5 | 4.0 ± 0.5 |
| *Lactobacillus brevis* | 8.5 ± 0.4 | 7.0 ± 0.5 | 6.8 ± 0.3 | 6.5 ± 0.4 | 5.0 ± 0.8 | 4.0 ± 0.7 | 4.0 ± 0.5 | 3.0 ± 0.5 | 3.0 ± 0.6 |
| *Lactobacillus oris* | 8.6 ± 0.4 | 7.5 ± 0.4 | 6.0 ± 0.5 | 6.0 ± 0.3 | 5.5 ± 0.7 | 5.5 ± 0.3 | 4.5 ± 0.4 | 4.0 ± 0.8 | 3.5 ± 0.4 |
| *Lactobacillus vaginalis* | 9.5 ± 0.3 | 9.5 ± 0.5 | 9.0 ± 0.3 | 9.0 ± 0.4 | 8.5 ± 0.6 | 8.0 ± 0.7 | 7.5 ± 0.8 | 7.5 ± 0.7 | 5.0 ± 0.4 |
| *Lactobacillus gasseri* | 7.6 ± 0.5 | 7.4 ± 0.5 | 7.0 ± 0.5 | 6.5 ± 0.3 | 6.0 ± 0.4 | 6.0 ± 0.6 | 5.5 ± 0.4 | 5.0 ± 0.7 | 4.5 ± 0.8 |

**02a.** Effect of pH on the antagonistic activity of extracted bacteriocins of *L. plantarum* against different bacterial pathogens. Inhibition zone were measured as mm ± Standard deviation.

| **Indicator strains** | **Bacteriocin producing *L. plantarum*** | | | | | | | | |
|---|---|---|---|---|---|---|---|---|---|
| | **pH 3** | **pH 4** | **pH 5** | **pH 6** | **pH 7** | **pH 8** | **pH 9** | **pH 10** | **pH 11** |
| *Bacillus cereus* | 4.5 ± 0.5 | 5.4 ± 0.0 | 7.6 ± 0.3 | 8.0 ± 0.3 | 8.4 ± 0.5 | 6.5 ± 0.5 | 5.0 ± 0.5 | 4.0 ± 0.5 | 3.3 ± 0.5 |
| *Bacillus subtilis* | 4.4 ± 0.3 | 5.0 ± 0.5 | 7.5 ± 0.3 | 7.8 ± 0.4 | 7.0 ± 0.3 | 6.0 ± 0.3 | 4.0 ± 0.3 | 4.2 ± 0.3 | 3.0 ± 0.3 |
| *Staphylococcus aureus* | 5.3 ± 0.3 | 4.5 ± 0.4 | 6.5 ± 0.5 | 7.0 ± 0.5 | 7.3 ± 0.3 | 5.0 ± 0.9 | 4.5 ± 0.3 | 4.0 ± 0.8 | 3.5 ± 0.7 |
| *Staphylococcus epidermidis* | 4.0 ± 0.6 | 4.8 ± 0.3 | 6.0 ± 0.5 | 6.8 ± 0.3 | 7.3 ± 0.5 | 5.0 ± 0.5 | 4.3 ± 0.5 | 3.3 ± 0.5 | 3.0 ± 0.5 |
| *Enterococcus faecalis* | - | - | - | - | - | - | - | - | - |
| *Staphylococcus faecalis* | 5.5 ± 0.5 | 6.0 ± 0.5 | 6.5 ± 0.5 | 6.8 ± 0.5 | 6.8 ± 0.5 | 5.2 ± 0.5 | 4.2 ± 0.5 | 4.0 ± 0.5 | 3.5 ± 0.5 |
| *Listeria monocytogenes* | 5.5 ± 0.7 | 7.0 ± 0.6 | 9.5 ± 0.3 | 9.2 ± 0.4 | 8.5 ± 0.5 | 7.0 ± 0.6 | 5.0 ± 0.3 | 4.5 ± 0.4 | 3.0 ± 0.4 |
| *Streptococcus thermophilus* | 5.0 ± 0.5 | 6.0 ± 0.6 | 8.3 ± 0.3 | 8.0 ± 0.5 | 7.5 ± 0.4 | 4.0 ± 0.7 | 3.2 ± 0.8 | 3.0 ± 0.5 | 2.4 ± 0.5 |
| *Clostridium spp.* | 5.3 ± 0.4 | 5.5 ± 0.5 | 6.8 ± 0.3 | 7.4 ± 0.4 | 7.6 ± 0.5 | 5.0 ± 0.4 | 4.3 ± 0.9 | 3.5 ± 0.8 | 3.5 ± 0.4 |
| *Pseudomonas aeruginosa* | - | - | - | - | - | - | - | - | - |
| *Vibrio parahemolyticus* | 4.0 ± 0.5 | 4.5 ± 0.3 | 5.5 ± 0.5 | 5.2 ± 0.3 | 5.0 ± 0.6 | 4.0 ± 0.8 | 3.3 ± 0.4 | 3.0 ± 0.9 | 3.0 ± 0.6 |
| *Salmonella typhi* | 4.5 ± 0.3 | 4.5 ± 0.7 | 5.3 ± 0.3 | 5.0 ± 0.3 | 5.0 ± 0.3 | 3.5 ± 0.5 | 3.5 ± 0.6 | 2.5 ± 0.3 | 2.2 ± 0.7 |
| *Shigella flexineri* | 3.5 ± 0.3 | 4.2 ± 0.5 | 5.0 ± 0.8 | 5.4 ± 0.9 | 4.5 ± 0.6 | 3.3 ± 0.4 | 3.0 ± 0.5 | 3.0 ± 0.5 | 2.5 ± 0.9 |
| *Escherichia coli* | 3.3 ± 0.5 | 4.0 ± 0.9 | 5.5 ± 0.5 | 5.4 ± 0.3 | 5.0 ± 0.5 | 4.4 ± 0.6 | 4.0 ± 0.5 | 4.0 ± 0.3 | 3.2 ± 0.5 |
| *Klebsiella spp.* | 4.2 ± 0.5 | 4.5 ± 0.3 | 5.2 ± 0.3 | 6.0 ± 0.5 | 6.6 ± 0.3 | 5.2 ± 0.6 | 4.0 ± 0.4 | 3.0 ± 0.9 | 2.3 ± 0.3 |
| *Serratia marcescens* | 6.0 ± 0.5 | 6.2 ± 0.9 | 6.5 ± 0.7 | 7.0 ± 0.5 | 7.3 ± 0.6 | 5.5 ± 0.8 | 4.5 ± 0.5 | 3.5 ± 0.5 | 3.0 ±0.4 |
| *Lactobacillus acidophilus* | 5.5 ± 0.5 | 5.5 ± 0.8 | 7.3 ± 0.5 | 7.6 ± 0.5 | 7.5 ± 0.6 | 7.2 ± 0.5 | 6.0 ± 0.9 | 5.5 ± 0.5 | 4.0 ± 0.7 |
| *Lactobacillus brevis* | 6.5 ± 0.3 | 7.0 ± 0.5 | 7.6 ± 0.4 | 8.0 ± 0.3 | 8.0 ± 0.8 | 6.0 ± 0.5 | 4.5 ± 0.7 | 3.3 ± 0.6 | 3.0 ± 0.5 |
| *Lactobacillus oris* | 6.5 ± 0.4 | 7.0 ± 0.3 | 8.5 ± 0.5 | 7.4 ± 0.5 | 7.0 ± 0.9 | 5.0 ± 0.3 | 4.3 ± 0.7 | 4.0 ± 0.8 | 3.7 ± 0.9 |
| *Lactobacillus vaginalis* | 6.0 ± 0.5 | 6.8 ± 0.5 | 9.0 ± 0.4 | 8.5 ± 0.3 | 8.0 ± 0.6 | 7.3 ± 0.9 | 7.0 ± 0.8 | 7.0 ± 0.6 | 5.5 ± 0.5 |
| *Lactobacillus gasseri* | 6.0 ± 0.5 | 7.0 ± 0.5 | 8.0 ± 0.5 | 8.3 ± 0.3 | 8.0 ± 0.4 | 6.8 ± 0.6 | 5.4 ± 0.4 | 5.0 ± 0.7 | 4.3 ± 0.8 |

**02b.** Effect of pH on the antagonistic activity of extracted bacteriocins of *L. paracasei ss paracasei* against different bacterial pathogens. Inhibition zone were measured as mm ± Standard deviation.

| **Indicator strains** | **Bacteriocin producing *L. paracasei ss paracasei*** | | | | | | | | |
|---|---|---|---|---|---|---|---|---|---|
| | **pH 3** | **pH 4** | **pH 5** | **pH 6** | **pH 7** | **pH 8** | **pH 9** | **pH 10** | **pH 11** |
| *Bacillus cereus* | 4.0 ± 0.3 | 5.5 ± 0.5 | 7.0 ± 0.5 | 7.5 ± 0.3 | 8.8 ± 0.4 | 6.0 ± 0.7 | 5.5 ± 0.3 | 4.5 ± 0.7 | 3.0 ± 0.5 |
| *Bacillus subtilis* | 4.0 ± 0.4 | 4.8 ± 0.5 | 6.5 ± 0.5 | 6.8 ± 0.3 | 7.2 ± 0.4 | 6.5 ± 0.3 | 5.0 ± 0.8 | 4.5 ± 0.6 | 2.5 ± 0.5 |
| *Staphylococcus aureus* | 5.0 ± 0.3 | 5.5 ± 0.4 | 6.5 ± 0.5 | 7.0 ± 0.5 | 7.5 ± 0.3 | 5.5 ± 0.5 | 4.5 ± 0.6 | 4.0 ± 0.7 | 3.0 ± 0.8 |
| *Staphylococcus epidermidis* | 3.0 ± 0.3 | 3.5 ± 0.5 | 4.0 ± 0.6 | 4.5 ± 0.7 | 6.0 ± 0.8 | 5.5 ± 0.9 | 4.4 ± 0.3 | 3.0 ± 0.5 | 3.0 ± 0.6 |
| *Enterococcus faecalis* | - | - | - | - | - | - | - | - | - |
| *Staphylococcus faecalis* | 5.0 ± 0.3 | 6.5 ± 0.5 | 6.5 ± 0.3 | 7.0 ± 0.4 | 7.0 ± 0.3 | 5.6 ± 0.6 | 4.5 ± 0.5 | 4.0 ± 0.7 | 3.5 ± 0.5 |
| *Listeria monocytogenes* | 6.0 ± 0.5 | 6.5 ± 0.4 | 8.0 ± 0.3 | 9.0 ± 0.6 | 8.5 ± 0.7 | 6.6 ± 0.6 | 5.0 ± 0.9 | 4.0 ± 0.4 | 2.5 ± 0.5 |
| *Streptococcus thermophilus* | 4.5 ± 0.3 | 5.0 ± 0.4 | 6.5 ± 0.6 | 7.2 ± 0.6 | 7.5 ± 0.5 | 4.0 ± 0.3 | 3.2 ± 0.7 | 3.0 ± 0.6 | 2.5 ± 0.5 |
| *Clostridium spp.* | 5.0 ± 0.5 | 5.5 ± 0.4 | 6.0 ± 0.4 | 7.0 ± 0.3 | 7.0 ± 0.6 | 4.5 ± 0.4 | 4.0 ± 0.7 | 3.5 ± 0.8 | 3.0 ± 0.4 |
| *Pseudomonas aeruginosa* | - | - | - | - | - | - | - | - | - |
| *Vibrio parahemolyticus* | 4.5 ± 0.3 | 5.0 ± 0.5 | 5.5 ± 0.6 | 5.0 ± 0.7 | 5.0 ± 0.6 | 4.0 ± 0.8 | 3.0 ± 0.4 | 3.0 ± 0.6 | 3.0 ± 0.7 |
| *Salmonella typhi* | 5.0 ± 0.3 | 5.0 ± 0.7 | 5.2 ± 0.3 | 5.0 ± 0.3 | 4.5 ± 0.3 | 3.5 ± 0.5 | 3.5 ± 0.6 | 2.5 ± 0.3 | 2.0 ± 0.7 |
| *Shigella flexineri* | 3.5 ± 0.3 | 4.0 ± 0.5 | 4.8 ± 0.5 | 5.0 ± 0.6 | 4.5 ± 0.6 | 3.5 ± 0.5 | 3.0 ± 0.4 | 3.0 ± 0.5 | 2.8 ± 0.3 |
| *Escherichia coli* | 3.5 ± 0.5 | 4.0 ± 0.3 | 5.2 ± 0.4 | 5.0 ± 0.3 | 5.0 ± 0.6 | 4.5 ± 0.5 | 4.0 ± 0.7 | 4.0 ± 0.3 | 3.5 ± 0.5 |
| *Klebsiella spp.* | 4.0 ± 0.5 | 4.2 ± 0.3 | 5.0 ± 0.5 | 6.0 ± 0.3 | 6.5 ± 0.3 | 5.0 ± 0.4 | 4.0 ± 0.4 | 3.0 ± 0.6 | 2.3 ± 0.5 |
| *Serratia marcescens* | 5.0 ± 0.5 | 5.4 ± 0.3 | 6.0 ± 0.5 | 6.5 ± 0.7 | 7.0 ± 0.6 | 5.5 ± 0.8 | 4.5 ± 0.5 | 3.5 ± 0.6 | 3.0 ±0.4 |
| *Lactobacillus acidophilus* | 5.5 ± 0.6 | 6.5 ± 0.4 | 7.3 ± 0.5 | 7.6 ± 0.5 | 7.0 ± 0.3 | 7.0 ± 0.5 | 6.0 ± 0.5 | 5.5 ± 0.5 | 4.0 ± 0.6 |
| *Lactobacillus brevis* | 6.5 ± 0.5 | 7.0 ± 0.3 | 7.6 ± 0.3 | 8.0 ± 0.4 | 8.0 ± 0.6 | 6.0 ± 0.5 | 4.5 ± 0.5 | 3.3 ± 0.6 | 3.0 ± 0.7 |
| *Lactobacillus oris* | 6.0 ± 0.4 | 6.5 ± 0.3 | 7.5 ± 0.3 | 7.0 ± 0.5 | 7.0 ± 0.7 | 5.0 ± 0.4 | 4.5 ± 0.6 | 4.0 ± 0.8 | 3.5 ± 0.6 |
| *Lactobacillus vaginalis* | 6.5 ± 0.4 | 6.8 ± 0.5 | 9.0 ± 0.4 | 8.5 ± 0.3 | 8.0 ± 0.5 | 7.5 ± 0.6 | 7.0 ± 0.6 | 6.0 ± 0.7 | 5.5 ± 0.5 |
| *Lactobacillus gasseri* | 5.5 ± 0.3 | 7.0 ± 0.3 | 8.0 ± 0.5 | 8.5 ± 0.3 | 8.0 ± 0.5 | 7.8 ± 0.6 | 6.4 ± 0.5 | 5.0 ± 0.7 | 4.3 ± 0.7 |

**02c.** Effect of pH on the antagonistic activity of extracted bacteriocins of *L. rhamnosus* against different bacterial pathogens. Inhibition zone were measured as mm ± Standard deviation.

| **Indicator strains** | **Bacteriocin producing *L. rhamnosus*** | | | | | | | | |
|---|---|---|---|---|---|---|---|---|---|
| | **pH 3** | **pH 4** | **pH 5** | **pH 6** | **pH 7** | **pH 8** | **pH 9** | **pH 10** | **pH 11** |
| *Bacillus cereus* | 4.8 ± 0.5 | 5.5 ± 0.3 | 7.5 ± 0.3 | 7.5 ± 0.5 | 8.0 ± 0.5 | 6.5 ± 0.3 | 6.0 ± 0.4 | 5.0 ± 0.5 | 3.0 ± 0.6 |
| *Bacillus subtilis* | 4.5 ± 0.4 | 5.0 ± 0.5 | 7.0 ± 0.3 | 7.5 ± 0.4 | 7.2 ± 0.5 | 6.0 ± 0.3 | 5.0 ± 0.3 | 4.5 ± 0.5 | 3.0 ± 0.3 |
| *Staphylococcus aureus* | 5.5 ± 0.5 | 5.0 ± 0.4 | 6.0 ± 0.3 | 6.5 ± 0.5 | 7.0 ± 0.3 | 5.5 ± 0.7 | 4.0 ± 0.3 | 4.0 ± 0.5 | 3.5 ± 0.6 |
| *Staphylococcus epidermidis* | 4.0 ± 0.6 | 4.5 ± 0.7 | 5.5 ± 0.5 | 6.0 ± 0.6 | 7.5 ± 0.5 | 5.0 ± 0.3 | 4.5 ± 0.5 | 3.0 ± 0.3 | 3.0 ± 0.5 |
| *Enterococcus faecalis* | - | - | - | - | - | - | - | - | - |
| *Staphylococcus faecalis* | 5.5 ± 0.3 | 5.8 ± 0.5 | 6.5 ± 0.3 | 7.0 ± 0.5 | 7.0 ± 0.7 | 6.2 ± 0.5 | 5.2 ± 0.4 | 4.0 ± 0.5 | 3.5 ± 0.6 |
| *Listeria monocytogenes* | 5.6 ± 0.7 | 7.8 ± 0.6 | 11.0 ± 0.3 | 10.5 ± 0.4 | 8.0 ± 0.5 | 5.5 ± 0.6 | 4.2 ± 0.3 | 4.0 ± 0.4 | 2.8 ± 0.4 |
| *Streptococcus thermophilus* | 5.5 ± 0.5 | 6.0 ± 0.6 | 7.5 ± 0.5 | 7.5 ± 0.3 | 7.0 ± 0.4 | 5.0 ± 0.7 | 4.5 ± 0.6 | 4.0 ± 0.4 | 2.5 ± 0.5 |
| *Clostridium spp.* | 5.0 ± 0.4 | 5.5 ± 0.5 | 6.5 ± 0.3 | 7.0 ± 0.4 | 7.0 ± 0.5 | 6.0 ± 0.4 | 4.5 ± 0.9 | 3.5 ± 0.8 | 3.0 ± 0.4 |
| *Pseudomonas aeruginosa* | - | - | - | - | - | - | - | - | - |
| *Vibrio parahemolyticus* | 4.0 ± 0.5 | 4.8 ± 0.5 | 6.0 ± 0.3 | 5.5 ± 0.3 | 5.0 ± 0.6 | 4.5 ± 0.4 | 3.3 ± 0.4 | 3.0 ± 0.7 | 3.0 ± 0.9 |
| *Salmonella typhi* | 4.0 ± 0.3 | 4.5 ± 0.4 | 5.2 ± 0.3 | 5.0 ± 0.5 | 5.0 ± 0.3 | 4.5 ± 0.5 | 3.5 ± 0.7 | 2.5 ± 0.6 | 2.5 ± 0.5 |
| *Shigella flexineri* | 3.5 ± 0.3 | 4.5 ± 0.5 | 5.0 ± 0.4 | 5.5 ± 0.3 | 4.5 ± 0.6 | 3.5 ± 0.5 | 3.0 ± 0.5 | 3.0 ± 0.7 | 2.5 ± 0.9 |
| *Escherichia coli* | 3.5 ± 0.5 | 4.0 ± 0.6 | 5.5 ± 0.7 | 5.4 ± 0.3 | 5.0 ± 0.4 | 4.5 ± 0.6 | 4.0 ± 0.3 | 4.0 ± 0.3 | 3.0 ± 0.5 |
| *Klebsiella spp.* | 4.0 ± 0.5 | 4.5 ± 0.3 | 5.0 ± 0.3 | 6.0 ± 0.6 | 6.5 ± 0.3 | 5.2 ± 0.7 | 4.5 ± 0.4 | 3.0 ± 0.5 | 2.5 ± 0.3 |
| *Serratia marcescens* | 4.0 ± 0.5 | 5.2 ± 0.3 | 6.0 ± 0.6 | 6.0 ± 0.5 | 6.3 ± 0.6 | 5.0 ± 0.7 | 4.5 ± 0.5 | 3.5 ± 0.4 | 3.0 ±0.3 |
| *Lactobacillus acidophilus* | 5.0 ± 0.3 | 5.5 ± 0.8 | 7.0 ± 0.5 | 7.5 ± 0.9 | 7.5 ± 0.7 | 7.0 ± 0.5 | 6.0 ± 0.9 | 5.5 ± 0.5 | 4.0 ± 0.7 |
| *Lactobacillus brevis* | 6.0 ± 0.5 | 6.5 ± 0.5 | 7.0 ± 0.3 | 7.5 ± 0.3 | 8.0 ± 0.4 | 6.0 ± 0.5 | 4.5 ± 0.5 | 3.5 ± 0.6 | 3.0 ± 0.7 |
| *Lactobacillus oris* | 6.5 ± 0.4 | 7.5 ± 0.3 | 8.5 ± 0.5 | 7.0 ± 0.5 | 7.0 ± 0.3 | 5.5 ± 0.5 | 4.5 ± 0.7 | 4.0 ± 0.4 | 3.7 ± 0.5 |
| *Lactobacillus vaginalis* | 6.0 ± 0.5 | 6.5 ± 0.3 | 8.5 ± 0.4 | 8.0 ± 0.5 | 7.5 ± 0.6 | 7.0 ± 0.7 | 7.0 ± 0.4 | 6.0 ± 0.3 | 5.5 ± 0.5 |
| *Lactobacillus gasseri* | 6.0 ± 0.3 | 7.0 ± 0.5 | 7.5 ± 0.5 | 8.4 ± 0.3 | 8.0 ± 0.5 | 6.0 ± 0.6 | 5.5 ± 0.4 | 5.0 ± 0.3 | 4.5 ± 0.7 |

**02d.** Effect of pH on the antagonistic activity of extracted bacteriocins of C4: *L. farciminis* against different bacterial pathogens. Inhibition zone were measured as mm ± Standard deviation.

| **Indicator strains** | **Bacteriocin producing *L. farciminis*** | | | | | | | | |
|---|---|---|---|---|---|---|---|---|---|
| | **pH 3** | **pH 4** | **pH 5** | **pH 6** | **pH 7** | **pH 8** | **pH 9** | **pH 10** | **pH 11** |
| *Bacillus cereus* | 4.5 ± 0.5 | 5.5 ± 0.3 | 6.0 ± 0.6 | 7.0 ± 0.3 | 7.5 ± 0.5 | 6.0 ± 0.4 | 5.0 ± 0.5 | 4.0 ± 0.7 | 3.0 ± 0.5 |
| *Bacillus subtilis* | 4.0 ± 0.5 | 5.2 ± 0.5 | 6.5 ± 0.7 | 7.2 ± 0.4 | 7.0 ± 0.6 | 6.5 ± 0.3 | 4.5 ± 0.4 | 4.5 ± 0.3 | 3.5 ± 0.5 |
| *Staphylococcus aureus* | 5.5 ± 0.3 | 5.5 ± 0.5 | 6.5 ± 0.5 | 7.0 ± 0.7 | 7.2 ± 0.3 | 5.5 ± 0.5 | 4.5 ± 0.3 | 4.0 ± 0.3 | 3.0 ± 0.4 |
| *Staphylococcus epidermidis* | 4.5 ± 0.3 | 5.8 ± 0.6 | 6.0 ± 0.5 | 6.5 ± 0.5 | 7.0 ± 0.7 | 5.0 ± 0.5 | 4.2 ± 0.7 | 3.0 ± 0.5 | 3.0 ± 0.4 |
| *Enterococcus faecalis* | - | - | - | - | - | - | - | - | - |
| *Staphylococcus faecalis* | 5.0 ± 0.3 | 6.0 ± 0.5 | 6.0 ± 0.5 | 6.5 ± 0.6 | 6.0 ± 0.5 | 5.0 ± 0.3 | 4.2 ± 0.5 | 4.0 ± 0.5 | 3.0 ± 0.4 |
| *Listeria monocytogenes* | 5.0 ± 0.4 | 7.0 ± 0.6 | 10.0 ± 0.5 | 11.0 ± 0.4 | 8.0 ± 0.3 | 6.5 ± 0.6 | 4.0 ± 0.5 | 4.0 ± 0.4 | 2.5 ± 0.5 |
| *Streptococcus thermophilus* | 5.5 ± 0.3 | 6.0 ± 0.4 | 7.0 ± 0.3 | 7.0 ± 0.7 | 7.5 ± 0.4 | 4.5 ± 0.3 | 3.0 ± 0.6 | 3.0 ± 0.5 | 2.4 ± 0.3 |
| *Clostridium spp.* | 5.6 ± 0.5 | 5.0 ± 0.4 | 6.0 ± 0.3 | 7.2 ± 0.5 | 7.5 ± 0.5 | 5.0 ± 0.4 | 4.0 ± 0.7 | 3.5 ± 0.6 | 3.0 ± 0.5 |
| *Pseudomonas aeruginosa* | - | - | - | - | - | - | - | - | - |
| *Vibrio parahemolyticus* | 4.5 ± 0.5 | 4.5 ± 0.3 | 5.2 ± 0.5 | 5.0 ± 0.3 | 5.0 ± 0.7 | 4.0 ± 0.8 | 3.0 ± 0.6 | 3.0 ± 0.4 | 2.5 ± 0.6 |
| *Salmonella typhi* | 4.5 ± 0.3 | 4.5 ± 0.5 | 5.0 ± 0.4 | 5.0 ± 0.5 | 4.5 ± 0.3 | 4.0 ± 0.7 | 3.5 ± 0.6 | 3.5 ± 0.3 | 3.0 ± 0.5 |
| *Shigella flexineri* | 4.0 ± 0.5 | 4.0 ± 0.6 | 4.5 ± 0.8 | 5.2 ± 0.7 | 4.0 ± 0.4 | 4.0 ± 0.6 | 3.0 ± 0.5 | 3.0 ± 0.9 | 2.5 ± 0.5 |
| *Escherichia coli* | 3.5 ± 0.3 | 4.2 ± 0.6 | 5.5 ± 0.5 | 5.2 ± 0.3 | 5.0 ± 0.4 | 4.5 ± 0.6 | 4.0 ± 0.5 | 4.0 ± 0.6 | 3.0 ± 0.7 |
| *Klebsiella spp.* | 4.2 ± 0.5 | 4.5 ± 0.6 | 5.2 ± 0.3 | 6.0 ± 0.5 | 6.5 ± 0.7 | 5.0 ± 0.6 | 4.0 ± 0.4 | 3.0 ± 0.6 | 2.5 ± 0.5 |
| *Serratia marcescens* | 5.5 ± 0.5 | 6.0 ± 0.3 | 6.5 ± 0.7 | 7.0 ± 0.6 | 7.0 ± 0.4 | 5.5 ± 0.8 | 4.5 ± 0.5 | 3.5 ± 0.5 | 3.0 ±0.7 |
| *Lactobacillus acidophilus* | 5.0 ± 0.3 | 6.5 ± 0.8 | 7.2 ± 0.7 | 7.8 ± 0.5 | 7.5 ± 0.4 | 7.2 ± 0.5 | 6.5 ± 0.3 | 5.5 ± 0.5 | 4.0 ± 0.6 |
| *Lactobacillus brevis* | 6.0 ± 0.3 | 7.0 ± 0.5 | 7.5 ± 0.4 | 7.5 ± 0.3 | 8.0 ± 0.6 | 6.5 ± 0.5 | 4.5 ± 0.5 | 3.5 ± 0.6 | 3.0 ± 0.7 |
| *Lactobacillus oris* | 6.5 ± 0.4 | 7.0 ± 0.3 | 7.5 ± 0.5 | 7.5 ± 0.4 | 7.0 ± 0.5 | 5.5 ± 0.3 | 4.5 ± 0.7 | 4.0 ± 0.6 | 3.5 ± 0.9 |
| *Lactobacillus vaginalis* | 6.0 ± 0.5 | 6.5 ± 0.5 | 8.0 ± 0.4 | 8.0 ± 0.3 | 7.0 ± 0.8 | 7.0 ± 0.9 | 5.0 ± 0.5 | 5.0 ± 0.6 | 4.5 ± 0.7 |
| *Lactobacillus gasseri* | 6.0 ± 0.5 | 7.0 ± 0.4 | 7.0 ± 0.5 | 8.0 ± 0.3 | 8.0 ± 0.4 | 6.8 ± 0.6 | 5.4 ± 0.7 | 5.0 ± 0.4 | 4.5 ± 0.5 |

**02e.** Effect of pH on the antagonistic activity of extracted bacteriocins of *L. bifermentans* against different bacterial pathogens. Inhibition zone were measured as mm ± Standard deviation.

| Indicator strains | Bacteriocin producing *L. bifermentans* | | | | | | | | |
|---|---|---|---|---|---|---|---|---|---|
| | **pH 3** | **pH 4** | **pH 5** | **pH 6** | **pH 7** | **pH 8** | **pH 9** | **pH 10** | **pH 11** |
| *Bacillus cereus* | 4.5 ± 0.5 | 5.4 ± 0.3 | 7.5 ± 0.4 | 8.0 ± 0.3 | 8.5 ± 0.5 | 6.5 ± 0.5 | 5.0 ± 0.7 | 4.5 ± 0.5 | 3.5 ± 0.8 |
| *Bacillus subtilis* | 4.0 ± 0.3 | 5.2 ± 0.5 | 7.0 ± 0.3 | 7.0 ± 0.4 | 6.0 ± 0.7 | 6.0 ± 0.3 | 4.5 ± 0.4 | 4.2 ± 0.3 | 3.0 ± 0.5 |
| *Staphylococcus aureus* | 5.0 ± 0.3 | 4.8 ± 0.4 | 5.5 ± 0.5 | 6.0 ± 0.5 | 7.0 ± 0.4 | 5.0 ± 0.9 | 4.5 ± 0.3 | 4.0 ± 0.5 | 3.5 ± 0.7 |
| *Staphylococcus epidermidis* | 4.0 ± 0.6 | 4.5 ± 0.3 | 5.0 ± 0.5 | 6.8 ± 0.3 | 7.0 ± 0.7 | 5.5 ± 0.5 | 4.2 ± 0.4 | 3.5 ± 0.5 | 3.0 ± 0.3 |
| *Enterococcus faecalis* | - | - | - | - | - | - | - | - | - |
| *Staphylococcus faecalis* | 5.5 ± 0.3 | 6.5 ± 0.5 | 6.5 ± 0.8 | 7.2 ± 0.5 | 7.2 ± 0.7 | 5.5 ± 0.5 | 4.5 ± 0.5 | 4.0 ± 0.4 | 3.5 ± 0.3 |
| *Listeria monocytogenes* | 5.0 ± 0.5 | 6.0 ± 0.6 | 9.5 ± 0.6 | 10.0 ± 0.4 | 8.5 ± 0.3 | 6.2 ± 0.6 | 4.5 ± 0.3 | 3.5 ± 0.7 | 2.5 ± 0.4 |
| *Streptococcus thermophilus* | 5.0 ± 0.6 | 5.5 ± 0.5 | 7.4 ± 0.5 | 8.0 ± 0.3 | 7.0 ± 0.4 | 5.5 ± 0.7 | 3.5 ± 0.8 | 3.0 ± 0.4 | 2.5 ± 0.5 |
| *Clostridium spp.* | 5.0 ± 0.4 | 6.5 ± 0.5 | 6.5 ± 0.3 | 7.0 ± 0.3 | 7.6 ± 0.5 | 5.5 ± 0.8 | 4.3 ± 0.9 | 3.5 ± 0.4 | 3.5 ± 0.4 |
| *Pseudomonas aeruginosa* | - | - | - | - | - | - | - | - | - |
| *Vibrio parahemolyticus* | 4.0 ± 0.5 | 4.5 ± 0.3 | 4.5 ± 0.5 | 5.0 ± 0.5 | 5.0 ± 0.6 | 4.5 ± 0.8 | 3.5 ± 0.4 | 3.0 ± 0.7 | 3.0 ± 0.6 |
| *Salmonella typhi* | 4.5 ± 0.3 | 4.5 ± 0.7 | 5.2 ± 0.5 | 5.0 ± 0.3 | 5.0 ± 0.6 | 4.5 ± 0.5 | 3.5 ± 0.7 | 2.5 ± 0.3 | 2.5 ± 0.5 |
| *Shigella flexineri* | 4.5 ± 0.5 | 4.0 ± 0.3 | 5.5 ± 0.8 | 5.2 ± 0.9 | 4.5 ± 0.8 | 3.5 ± 0.4 | 3.0 ± 0.6 | 3.0 ± 0.5 | 2.5 ± 0.7 |
| *Escherichia coli* | 3.5 ± 0.3 | 4.0 ± 0.7 | 5.5 ± 0.5 | 5.0 ± 0.4 | 5.0 ± 0.5 | 4.5 ± 0.7 | 4.0 ± 0.5 | 4.0 ± 0.5 | 3.0 ± 0.3 |
| *Klebsiella spp.* | 4.0 ± 0.6 | 4.5 ± 0.3 | 5.0 ± 0.3 | 6.5 ± 0.6 | 6.6 ± 0.3 | 5.5 ± 0.4 | 4.0 ± 0.4 | 3.0 ± 0.3 | 2.5 ± 0.5 |
| *Serratia marcescens* | 6.0 ± 0.3 | 6.5 ± 0.9 | 6.5 ± 0.8 | 7.0 ± 0.5 | 7.2 ± 0.6 | 5.5 ± 0.7 | 4.5 ± 0.5 | 3.5 ± 0.3 | 3.0 ±0.4 |
| *Lactobacillus acidophilus* | 5.0 ± 0.5 | 6.5 ± 0.4 | 7.2 ± 0.5 | 7.5 ± 0.7 | 7.5 ± 0.6 | 7.0 ± 0.5 | 6.0 ± 0.4 | 5.5 ± 0.5 | 4.0 ± 0.7 |
| *Lactobacillus brevis* | 6.0 ± 0.6 | 7.0 ± 0.5 | 7.5 ± 0.4 | 8.0 ± 0.3 | 7.0 ± 0.6 | 6.0 ± 0.5 | 5.5 ± 0.7 | 3.5 ± 0.5 | 3.0 ± 0.6 |
| *Lactobacillus oris* | 6.5 ± 0.4 | 7.5 ± 0.3 | 8.0 ± 0.6 | 7.5 ± 0.5 | 7.0 ± 0.9 | 5.0 ± 0.7 | 4.5 ± 0.8 | 4.0 ± 0.5 | 3.5 ± 0.5 |
| *Lactobacillus vaginalis* | 6.0 ± 0.5 | 6.5 ± 0.5 | 7.0 ± 0.4 | 8.5 ± 0.3 | 8.0 ± 0.6 | 7.2 ± 0.7 | 7.0 ± 0.8 | 7.0 ± 0.5 | 5.0 ± 0.6 |
| *Lactobacillus gasseri* | 6.5 ± 0.5 | 7.0 ± 0.6 | 8.0 ± 0.5 | 8.5 ± 0.3 | 8.0 ± 0.5 | 7.0 ± 0.6 | 5.5 ± 0.8 | 5.0 ± 0.7 | 4.5 ± 0.6 |

**03a.** Effect of NaCl on the antagonistic activity of extracted bacteriocins of *L. plantarum* against different bacterial pathogens. Inhibition zone were measured as mm ± Standard deviation.

| **Indicator strains** | **Bacteriocin producing *L.plantarum*** | | | | | | |
|---|---|---|---|---|---|---|---|
| | **1%** | **2%** | **3%** | **4%** | **5%** | **6%** | **7%** |
| *Bacillus cereus* | 8.0 ± 0.5 | 7.4 ± 0.9 | 7.2 ± 0.3 | 6.5 ± 0.3 | 6.0 ± 0.5 | 5.5 ± 0.8 | 5.0 ± 0.9 |
| *Bacillus subtilis* | 7.0 ± 0.3 | 7.0 ± 0.5 | 6.5 ± 0.3 | 6.3 ± 0.4 | 6.0 ± 0.3 | 6.0 ± 0.3 | 4.0 ± 0.3 |
| *Staphylococcus aureus* | 8.5 ± 0.3 | 7.3 ± 0.4 | 7.0 ±0.5 | 6.0 ± 0.5 | 5.0 ± 0.3 | 5.0 ± 0.3 | 4.5 ± 0.3 |
| *Staphylococcus epidermidis* | 7.5 ± 0.6 | 6.4 ± 0.3 | 6.0 ±0.5 | 5.8 ± 0.3 | 5.3 ± 0.5 | 5.0 ± 0.5 | 4.3 ± 0.5 |
| *Enterococcus faecalis* | - | - | - | - | - | - | - |
| *Staphylococcus faecalis* | 6.5 ± 0.5 | 6.0 ± 0.5 | 5.5 ± 0.5 | 4.5 ± 0.5 | 4.0 ± 0.5 | 3.2 ± 0.5 | 3.0 ± 0.5 |
| *Listeria monocytogenes* | 9.2 ± 0.7 | 7.5 ± 0.6 | 6.0 ± 0.3 | 4.8 ±0.4 | 4.5 ±0.5 | 5.5 ± 0.6 | 4.5 ± 0.3 |
| *Streptococcus thermophilus* | 6.0 ± 0.5 | 6.0 ± 0.6 | 5.3 ± 0.3 | 4.5 ± 0.5 | 4.5 ± 0.4 | 4.0 ± 0.7 | 3.2 ± 0.8 |
| *Clostridium spp.* | 6.5 ± 0.4 | 5.5 ± 0.5 | 4.8 ± 0.3 | 4.4 ± 0.4 | 4.0 ± 0.5 | 4.0 ± 0.4 | 3.0 ± 0.9 |
| *Pseudomonas aeruginosa* | - | - | - | - | - | - | - |
| *Vibrio parahemolyticus* | 6.5 ± 0.5 | 5.5 ± 0.3 | 5.3 ± 0.5 | 5.0 ± 0.3 | 4.0 ± 0.6 | 3.5 ± 0.8 | 3.3 ± 0.4 |
| *Salmonella typhi* | 5.5 ± 0.3 | 5.0 ± 0.7 | 4.3 ± 0.3 | 4.0 ± 0.3 | 4.0 ± 0.3 | 3.5 ± 0.5 | 3.0 ± 0.6 |
| *Shigella flexineri* | 4.5 ± 0.3 | 4.2 ± 0.5 | 4.0 ± 0.8 | 4.0 ± 0.9 | 3.5 ± 0.6 | 3.3 ± 0.4 | 2.3 ± 0.5 |
| *Escherichia coli* | 6.0 ± 0.5 | 5.0 ± 0.9 | 5.0 ± 0.5 | 4.4 ± 0.3 | 4.0 ± 0.5 | 3.0 ± 0.6 | 2.5 ± 0.5 |
| *Klebsiella spp.* | 5.0 ± 0.5 | 4.5 ± 0.3 | 4.2 ± 0.3 | 4.0 ± 0.5 | 3.5 ± 0.3 | 3.2 ± 0.6 | 3.0 ± 0.4 |
| *Serratia marcescens* | 6.5 ± 0.5 | 6.2 ± 0.9 | 6.0 ± 0.7 | 6.0 ± 0.5 | 5.4 ± 0.6 | 4.5 ± 0.8 | 3.0 ± 0.5 |
| *Lactobacillus acidophilus* | 8.5 ± 0.5 | 8.0 ± 0.8 | 8.3 ± 0.5 | 8.0 ± 0.5 | 7.5 ± 0.6 | 5.5 ± 0.5 | 4.0 ± 0.9 |
| *Lactobacillus brevis* | 9.0 ± 0.3 | 7.5 ± 0.5 | 6.0 ± 0.4 | 6.0 ± 0.3 | 5.0 ± 0.8 | 4.0 ± 0.5 | 3.0 ± 0.7 |
| *Lactobacillus oris* | 8.0 ± 0.4 | 7.0 ± 0.3 | 6.5 ± 0.5 | 6.0 ± 0.5 | 5.0 ± 0.9 | 4.0 ± 0.3 | 3.3 ± 0.7 |
| *Lactobacillus vaginalis* | 9.5 ± 0.5 | 8.0 ± 0.5 | 8.0 ± 0.4 | 7.0 ± 0.3 | 6.5 ± 0.6 | 5.3 ± 0.9 | 5.0 ± 0.8 |
| *Lactobacillus gasseri* | 8.0 ± 0.5 | 7.0 ± 0.5 | 5.5 ± 0.5 | 5.3 ± 0.3 | 4.0 ± 0.4 | 4.0 ± 0.6 | 3.4 ± 0.4 |

0**3b.** Effect of NaCl on the antagonistic activity of extracted bacteriocins of *L. paracasei ss paracasei* against different bacterial pathogens. Inhibition zone were measured as mm ± Standard deviation.

| **Indicator strains** | **Bacteriocin producing *L. paracasei ss paracasei*** | | | | | | |
|---|---|---|---|---|---|---|---|
| | **1%** | **2%** | **3%** | **4%** | **5%** | **6%** | **7%** |
| *Bacillus cereus* | 7.0 ± 0.6 | 6.4 ± 0.5 | 6.2 ± 0.3 | 5.5 ± 0.5 | 5.0 ± 0.5 | 5.0 ± 0.7 | 4.0± 0.6 |
| *Bacillus subtilis* | 8.0 ± 0.5 | 7.5 ± 0.6 | 6.5 ± 0.3 | 6.2 ± 0.4 | 6.0 ± 0.4 | 6.0 ± 0.3 | 4.0 ± 0.5 |
| *Staphylococcus aureus* | 8.0 ± 0.7 | 7.5 ± 0.4 | 7.0 ±0.5 | 6.0 ± 0.6 | 6.0 ± 0.3 | 5.0 ± 0.5 | 4.5 ± 0.3 |
| *Staphylococcus epidermidis* | 7.5 ± 0.5 | 6.0 ± 0.3 | 6.0 ±0.6 | 5.5 ± 0.3 | 5.2 ± 0.6 | 5.0 ± 0.5 | 4.0 ± 0.7 |
| *Enterococcus faecalis* | - | - | - | - | - | - | - |
| *Staphylococcus faecalis* | 6.5 ± 0.5 | 6.0 ± 0.7 | 5.5 ± 0.6 | 5.5 ± 0.4 | 4.2 ± 0.5 | 4.0 ± 0.3 | 3.0 ± 0.5 |
| *Listeria monocytogenes* | 9.5 ± 0.7 | 7.0 ± 0.6 | 5.6 ± 0.3 | 5.0 ±0.4 | 4.8 ±0.5 | 3.6 ± 0.6 | 2.0 ± 0.3 |
| *Streptococcus thermophilus* | 6.5 ± 0.5 | 6.5 ± 0.8 | 5.2 ± 0.3 | 4.5 ± 0.7 | 4.0 ± 0.4 | 4.0 ± 0.5 | 3.0 ± 0.6 |
| *Clostridium spp.* | 7.5 ± 0.4 | 6.5 ± 0.5 | 5.8 ± 0.8 | 4.5 ± 0.4 | 4.5 ± 0.9 | 4.0 ± 0.4 | 2.5 ± 0.7 |
| *Pseudomonas aeruginosa* | - | - | - | - | - | - | - |
| *Vibrio parahemolyticus* | 6.0 ± 0.5 | 5.5 ± 0.3 | 5.2 ± 0.6 | 5.0 ± 0.3 | 4.0 ± 0.5 | 3.5 ± 0.8 | 3.0 ± 0.5 |
| *Salmonella typhi* | 5.5 ± 0.5 | 5.5 ± 0.7 | 4.5 ± 0.3 | 4.0 ± 0.4 | 4.0 ± 0.3 | 3.5 ± 0.6 | 3.0 ± 0.4 |
| *Shigella flexineri* | 4.5 ± 0.5 | 4.5 ± 0.5 | 4.0 ± 0.6 | 3.5 ± 0.9 | 3.5 ± 0.4 | 3.0 ± 0.5 | 2.5 ± 0.5 |
| *Escherichia coli* | 6.5 ± 0.3 | 6.0 ± 0.9 | 5.0 ± 0.7 | 4.5 ± 0.3 | 4.0 ± 0.6 | 3.0 ± 0.5 | 3.0 ± 0.5 |
| *Klebsiella spp.* | 5.5 ± 0.5 | 4.5 ± 0.3 | 4.5 ± 0.8 | 4.0 ± 0.5 | 3.5 ± 0.7 | 3.5 ± 0.6 | 3.0 ± 0.5 |
| *Serratia marcescens* | 6.5 ± 0.5 | 6.0 ± 0.9 | 6.0 ± 0.4 | 5.5 ± 0.3 | 5.0 ± 0.6 | 4.5 ± 0.7 | 4.0 ± 0.6 |
| *Lactobacillus acidophilus* | 8.0 ± 0.5 | 7.0 ± 0.5 | 6.2 ± 0.3 | 6.0 ± 0.5 | 5.5 ± 0.7 | 5.5 ± 0.6 | 4.0 ± 0.5 |
| *Lactobacillus brevis* | 9.5 ± 0.3 | 8.5 ± 0.5 | 7.0 ± 0.5 | 7.0 ± 0.3 | 5.5 ± 0.5 | 4.5 ± 0.7 | 3.0 ± 0.5 |
| *Lactobacillus oris* | 8.0 ± 0.4 | 7.5 ± 0.3 | 6.5 ± 0.8 | 6.0 ± 0.5 | 5.0 ± 0.6 | 4.0 ± 0.4 | 3.5 ± 0.7 |
| *Lactobacillus vaginalis* | 9.0 ± 0.3 | 8.0 ± 0.5 | 8.0 ± 0.5 | 7.5 ± 0.3 | 6.5 ± 0.5 | 5.0 ± 0.6 | 5.0 ± 0.8 |
| *Lactobacillus gasseri* | 8.0 ± 0.5 | 6.0 ± 0.5 | 5.5 ± 0.7 | 5.5 ± 0.3 | 4.0 ± 0.6 | 4.0 ± 0.5 | 3.5 ± 0.5 |

**03c.** Effect of NaCl on the antagonistic activity of extracted bacteriocins of *L. rhamnosus* against different bacterial pathogens. Inhibition zone were measured as mm ± Standard deviation.

| **Indicator strains** | **Bacteriocin producing *L. rhamnosus*** | | | | | | |
|---|---|---|---|---|---|---|---|
| | **1%** | **2%** | **3%** | **4%** | **5%** | **6%** | **7%** |
| *Bacillus cereus* | 7.5 ± 0.5 | 7.5 ± 0.6 | 7.0 ± 0.3 | 6.5 ± 0.7 | 6.0 ± 0.5 | 5.0 ± 0.6 | 4.5 ± 0.5 |
| *Bacillus subtilis* | 7.0 ± 0.3 | 6.5 ± 0.7 | 6.5 ± 0.3 | 6.2 ± 0.4 | 6.0 ± 0.6 | 6.0 ± 0.3 | 4.0 ± 0.4 |
| *Staphylococcus aureus* | 7.5 ± 0.3 | 7.5 ± 0.8 | 6.0 ±0.4 | 6.0 ± 0.5 | 5.5 ± 0.6 | 5.5 ± 0.3 | 4.5 ± 0.5 |
| *Staphylococcus epidermidis* | 7.5 ± 0.7 | 6.5 ± 0.3 | 6.5 ±0.5 | 5.5 ± 0.6 | 5.0 ± 0.5 | 5.0 ± 0.4 | 4.0 ± 0.5 |
| *Enterococcus faecalis* | - | - | - | - | - | - | - |
| *Staphylococcus faecalis* | 6.0 ± 0.3 | 6.0 ± 0.5 | 5.5 ± 0.4 | 4.5 ± 0.5 | 4.5 ± 0.3 | 3.0 ± 0.5 | 3.0 ± 0.6 |
| *Listeria monocytogenes* | 10.0 ± 0.4 | 7.5 ± 0.5 | 5.5 ± 0.3 | 4.5 ±0.5 | 4.5 ±0.4 | 3.5 ± 0.3 | 2.8 ± 0.5 |
| *Streptococcus thermophilus* | 6.0 ± 0.5 | 6.0 ± 0.4 | 5.5 ± 0.3 | 5.0 ± 0.6 | 4.5 ± 0.5 | 4.0 ± 0.7 | 3.0 ± 0.6 |
| *Clostridium spp.* | 6.5 ± 0.4 | 6.5 ± 0.5 | 5.0 ± 0.5 | 5.2 ± 0.4 | 4.0 ± 0.6 | 4.0 ± 0.4 | 3.5 ± 0.7 |
| *Pseudomonas aeruginosa* | - | - | - | - | - | - | - |
| *Vibrio parahemolyticus* | 5.5 ± 0.3 | 5.5 ± 0.5 | 5.0 ± 0.5 | 5.0 ± 0.4 | 4.0 ± 0.6 | 4.0 ± 0.5 | 3.0 ± 0.6 |
| *Salmonella typhi* | 5.5 ± 0.5 | 5.5 ± 0.4 | 4.5 ± 0.3 | 4.5 ± 0.6 | 4.0 ± 0.3 | 3.5 ± 0.4 | 3.0 ± 0.5 |
| *Shigella flexineri* | 5.5 ± 0.3 | 4.5 ± 0.5 | 4.5 ± 0.4 | 4.0 ± 0.5 | 3.5 ± 0.6 | 3.5 ± 0.4 | 2.8 ± 0.7 |
| *Escherichia coli* | 6.0 ± 0.5 | 6.0 ± 0.7 | 5.0 ± 0.5 | 4.5 ± 0.5 | 4.5 ± 0.3 | 3.0 ± 0.6 | 2.5 ± 0.4 |
| *Klebsiella spp.* | 5.0 ± 0.5 | 5.0 ± 0.3 | 4.5 ± 0.4 | 4.0 ± 0.5 | 4.0 ± 0.6 | 3.5 ± 0.5 | 3.0 ± 0.4 |
| *Serratia marcescens* | 6.5 ± 0.3 | 6.5 ± 0.7 | 6.0 ± 0.5 | 6.0 ± 0.5 | 5.5 ± 0.8 | 5.5 ± 0.6 | 4.0 ± 0.4 |
| *Lactobacillus acidophilus* | 8.0 ± 0.5 | 8.0 ± 0.3 | 7.2 ± 0.5 | 7.0 ± 0.5 | 7.0 ± 0.4 | 5.0 ± 0.5 | 4.0 ± 0.8 |
| *Lactobacillus brevis* | 8.0 ± 0.5 | 7.0 ± 0.3 | 6.0 ± 0.4 | 6.0 ± 0.5 | 5.0 ± 0.7 | 4.0 ± 0.5 | 3.0 ± 0.6 |
| *Lactobacillus oris* | 7.0 ± 0.3 | 7.0 ± 0.3 | 6.5 ± 0.5 | 6.0 ± 0.3 | 5.0 ± 0.6 | 4.0 ± 0.7 | 4.0 ± 0.5 |
| *Lactobacillus vaginalis* | 9.0 ± 0.4 | 9.0 ± 0.5 | 8.0 ± 0.4 | 7.0 ± 0.5 | 6.5 ± 0.6 | 5.5 ± 0.7 | 5.0 ± 0.6 |
| *Lactobacillus gasseri* | 8.0 ± 0.3 | 7.0 ± 0.5 | 6.5 ± 0.3 | 6.5 ± 0.3 | 4.0 ± 0.6 | 4.0 ± 0.4 | 3.5 ± 0.3 |

**03d.** Effect of NaCl on the antagonistic activity of extracted bacteriocins of C4: *L. farciminis* against different bacterial pathogens. Inhibition zone were measured as mm ± Standard deviation.

| **Indicator strains** | **Bacteriocin producing *L. farciminis*** | | | | | | |
|---|---|---|---|---|---|---|---|
| | **1%** | **2%** | **3%** | **4%** | **5%** | **6%** | **7%** |
| *Bacillus cereus* | 7.0 ± 0.5 | 7.0 ± 0.6 | 6.5 ± 0.3 | 6.5 ± 0.5 | 6.0 ± 0.5 | 5.5 ± 0.7 | 4.0 ± 0.5 |
| *Bacillus subtilis* | 7.5 ± 0.3 | 7.5 ± 0.4 | 6.0 ± 0.3 | 6.0 ± 0.7 | 5.2 ± 0.3 | 4.5 ± 0.4 | 4.0 ± 0.3 |
| *Staphylococcus aureus* | 8.0 ± 0.4 | 7.2 ± 0.6 | 7.0 ±0.5 | 7.0 ± 0.7 | 5.5 ± 0.3 | 5.0 ± 0.4 | 4.5 ± 0.3 |
| *Staphylococcus epidermidis* | 7.5 ± 0.6 | 6.4 ± 0.3 | 6.0 ±0.5 | 5.8 ± 0.3 | 5.3 ± 0.5 | 5.0 ± 0.5 | 4.3 ± 0.5 |
| *Enterococcus faecalis* | - | - | - | - | - | - | - |
| *Staphylococcus faecalis* | 6.5 ± 0.7 | 6.5 ± 0.5 | 5.5 ± 0.6 | 5.5 ± 0.5 | 4.0 ± 0.3 | 4.0 ± 0.5 | 3.5 ± 0.4 |
| *Listeria monocytogenes* | 11.0 ± 0.7 | 7.8 ± 0.6 | 5.0 ± 0.3 | 4.0 ±0.4 | 4.5 ±0.5 | 3.3 ± 0.6 | 1.4 ± 0.3 |
| *Streptococcus thermophilus* | 6.5 ± 0.5 | 6.0 ± 0.6 | 6.0 ± 0.6 | 4.5 ± 0.5 | 4.2 ± 0.5 | 4.0 ± 0.7 | 3.0 ± 0.4 |
| *Clostridium spp.* | 7.5 ± 0.5 | 6.5 ± 0.5 | 5.8 ± 0.6 | 4.5 ± 0.3 | 4.0 ± 0.5 | 4.0 ± 0.4 | 2.5 ± 0.7 |
| *Pseudomonas aeruginosa* | - | - | - | - | - | - | - |
| *Vibrio parahemolyticus* | 6.0 ± 0.5 | 5.5 ± 0.6 | 5.5 ± 0.5 | 4.5 ± 0.5 | 4.0 ± 0.6 | 3.5 ± 0.8 | 3.0 ± 0.6 |
| *Salmonella typhi* | 5.0 ± 0.3 | 5.0 ± 0.5 | 4.5 ± 0.3 | 4.0 ± 0.4 | 4.0 ± 0.7 | 3.0 ± 0.6 | 3.0 ± 0.5 |
| *Shigella flexineri* | 5.0 ± 0.5 | 4.5 ± 0.5 | 4.0 ± 0.8 | 4.0 ± 0.7 | 3.5 ± 0.6 | 3.5 ± 0.5 | 2.5 ± 0.6 |
| *Escherichia coli* | 6.0 ± 0.5 | 5.0 ± 0.8 | 5.0 ± 0.5 | 4.5 ± 0.4 | 4.0 ± 0.3 | 3.0 ± 0.6 | 2.5 ± 0.7 |
| *Klebsiella spp.* | 5.0 ± 0.3 | 4.5 ± 0.3 | 4.5 ± 0.5 | 4.0 ± 0.5 | 3.5 ± 0.6 | 3.5 ± 0.7 | 3.0 ± 0.4 |
| *Serratia marcescens* | 6.0 ± 0.4 | 6.0 ± 0.3 | 5.2 ± 0.6 | 5.0 ± 0.5 | 4.5 ± 0.7 | 4.5 ± 0.8 | 3.0 ± 0.9 |
| *Lactobacillus acidophilus* | 8.0 ± 0.5 | 8.0 ± 0.4 | 7.2 ± 0.5 | 7.0 ± 0.3 | 7.0 ± 0.5 | 6.5 ± 0.5 | 5.0 ± 0.7 |
| *Lactobacillus brevis* | 7.0 ± 0.3 | 7.0 ± 0.7 | 6.0 ± 0.4 | 5.5 ± 0.4 | 5.0 ± 0.8 | 4.0 ± 0.7 | 3.5 ± 0.5 |
| *Lactobacillus oris* | 8.0 ± 0.4 | 7.0 ± 0.3 | 6.0 ± 0.3 | 6.0 ± 0.5 | 5.5 ± 0.7 | 4.0 ± 0.8 | 3.0 ± 0.5 |
| *Lactobacillus vaginalis* | 9.0 ± 0.3 | 8.5 ± 0.5 | 8.0 ± 0.4 | 7.0 ± 0.3 | 6.0 ± 0.5 | 5.0 ± 0.6 | 5.0 ± 0.7 |
| *Lactobacillus gasseri* | 7.0 ± 0.5 | 7.0 ± 0.6 | 5.5 ± 0.5 | 5.2 ± 0.3 | 4.5 ± 0.5 | 4.5 ± 0.6 | 3.5 ± 0.7 |

**03e.** Effect of NaCl on the antagonistic activity of extracted bacteriocins of *L. bifermentans* against different bacterial pathogens. Inhibition zone were measured as mm ± Standard deviation.

| **Indicator strains** | **Bacteriocin producing *L. bifermentans*** | | | | | | |
|---|---|---|---|---|---|---|---|
| | **1%** | **2%** | **3%** | **4%** | **5%** | **6%** | **7%** |
| *Bacillus cereus* | 7.5 ± 0.5 | 7.0 ± 0.5 | 7.0 ± 0.3 | 6.0 ± 0.3 | 6.0 ± 0.5 | 5.0 ± 0.7 | 5.0 ± 0.6 |
| *Bacillus subtilis* | 6.0 ± 0.5 | 6.0 ± 0.3 | 5.0 ± 0.3 | 4.3 ± 0.5 | 4.0 ± 0.3 | 4.0 ± 0.7 | 3.5 ± 0.6 |
| *Staphylococcus aureus* | 8.0 ± 0.5 | 7.5 ± 0.4 | 7.0 ±0.8 | 6.0 ± 0.5 | 5.0 ± 0.7 | 5.0 ± 0.3 | 4.5 ± 0.5 |
| *Staphylococcus epidermidis* | 7.0 ± 0.5 | 6.5 ± 0.3 | 6.0 ±0.4 | 5.8 ± 0.3 | 5.2 ± 0.3 | 5.2 ± 0.5 | 4.0 ± 0.6 |
| *Enterococcus faecalis* | - | - | - | - | - | - | - |
| *Staphylococcus faecalis* | 5.5 ± 0.3 | 5.0 ± 0.4 | 5.0 ± 0.5 | 4.5 ± 0.5 | 4.0 ± 0.3 | 4.0 ± 0.5 | 3.0 ± 0.4 |
| *Listeria monocytogenes* | 10.0 ± 0.7 | 8.0 ± 0.6 | 4.9 ± 0.3 | 4.0 ±0.4 | 4.4 ±0.5 | 3.2 ± 0.6 | 3.2 ± 0.3 |
| *Streptococcus thermophilus* | 6.0 ± 0.5 | 6.0 ± 0.4 | 5.5 ± 0.3 | 4.5 ± 0.7 | 4.0 ± 0.4 | 4.0 ± 0.6 | 3.5 ± 0.8 |
| *Clostridium spp.* | 6.0 ± 0.4 | 5.5 ± 0.3 | 4.8 ± 0.3 | 4.5 ± 0.5 | 4.0 ± 0.5 | 4.0 ± 0.4 | 3.5 ± 0.6 |
| *Pseudomonas aeruginosa* | - | - | - | - | - | - | - |
| *Vibrio parahemolyticus* | 5.5 ± 0.3 | 5.5 ± 0.3 | 5.2 ± 0.5 | 5.0 ± 0.5 | 4.0 ± 0.6 | 4.0 ± 0.7 | 3.0 ± 0.5 |
| *Salmonella typhi* | 5.0 ± 0.5 | 5.0 ± 0.3 | 4.5 ± 0.7 | 4.0 ± 0.5 | 4.0 ± 0.3 | 3.5 ± 0.4 | 3.0 ± 0.6 |
| *Shigella flexineri* | 5.5 ± 0.4 | 5.2 ± 0.5 | 5.0 ± 0.8 | 5.0 ± 0.7 | 4.5 ± 0.6 | 3.0 ± 0.5 | 2.5 ± 0.5 |
| *Escherichia coli* | 6.0 ± 0.4 | 5.5 ± 0.6 | 5.0 ± 0.7 | 4.5 ± 0.6 | 4.0 ± 0.5 | 3.0 ± 0.6 | 2.5 ± 0.5 |
| *Klebsiella spp.* | 5.0 ± 0.4 | 4.5 ± 0.3 | 4.2 ± 0.7 | 4.2 ± 0.5 | 4.0 ± 0.5 | 3.5 ± 0.6 | 3.0 ± 0.5 |
| *Serratia marcescens* | 6.0 ± 0.5 | 6.0 ± 0.4 | 5.0 ± 0.3 | 5.0 ± 0.5 | 4.5 ± 0.5 | 4.5 ± 0.6 | 3.0 ± 0.7 |
| *Lactobacillus acidophilus* | 8.0 ± 0.5 | 8.0 ± 0.8 | 7.5 ± 0.3 | 7.5 ± 0.5 | 7.0 ± 0.4 | 6.5 ± 0.5 | 5.0 ± 0.6 |
| *Lactobacillus brevis* | 8.0 ± 0.3 | 7.5 ± 0.6 | 6.0 ± 0.4 | 6.0 ± 0.4 | 5.5 ± 0.8 | 4.0 ± 0.7 | 3.5 ± 0.5 |
| *Lactobacillus oris* | 8.0 ± 0.4 | 7.0 ± 0.6 | 7.0 ± 0.5 | 6.0 ± 0.5 | 6.0 ± 0.8 | 4.0 ± 0.3 | 3.5 ± 0.4 |
| *Lactobacillus vaginalis* | 9.0 ± 0.3 | 8.0 ± 0.5 | 7.0 ± 0.6 | 7.0 ± 0.3 | 6.5 ± 0.5 | 5.2 ± 0.9 | 5.0 ± 0.3 |
| *Lactobacillus gasseri* | 8.0 ± 0.5 | 7.0 ± 0.5 | 5.0 ± 0.7 | 5.0 ± 0.3 | 4.5 ± 0.4 | 4.0 ± 0.5 | 3.5 ± 0.6 |
